# Nanoencapsulated *Syzygium aromaticum* oil alleviates acetic acid-induced ulcerative colitis in rats by influencing critical redox, NF-κB/iNOS, and Keap1/Nrf2/HO-1 signaling pathways

**DOI:** 10.1007/s10787-025-01939-z

**Published:** 2025-10-07

**Authors:** Gehad E. Elshopakey, Shaymaa Rezk, Ahmed Ateya, Tarek A. Elkhooly, Alaa A. Omar, Ali El-Far, Ekramy M. Elmorsy, Hala Magdy Anwer, Mona M. Elghareeb

**Affiliations:** 1 Department of Clinical Pathology, Faculty of Veterinary Medicine, Mansoura University, Mansoura, 35516 Egypt; 2 Department of Cytology and Histology, Faculty of Veterinary Medicine, Mansoura University, Mansoura, 35516 Egypt; 3 Department of Husbandry and Development of Animal Wealth, Faculty of Veterinary Medicine, Mansoura University, Mansoura, 35516 Egypt; 4 Nanomedicine Research Unit, Faculty of Medicine, Delta University for Science and Technology, Gamasa, 35712 Egypt; 5 Department of Refractories, Ceramics and Building Materials, National Research Centre, Giza, Egypt; 6 Key Laboratory of Epigenetics and Oncology, The Research Center for Preclinical Medicine, Southwest Medical University, Luzhou, 646000 China; 7 Center of Health Research, Northern Border University, 91431 Arar, Saudi Arabia; 8 Department of Physiology, Faculty of Medicine, Benha University, Benha, 13736 Egypt; 9 Department of Physiology, Faculty of Veterinary Medicine, Mansoura University, Mansoura, 35516 Egypt

**Keywords:** Ulcerative colitis, *Syzygium aromaticum* oil, Alginate/Chitosan, Polycaprolactone, NF-*κ*B/iNOS pathway, Keap1/Nrf2/HO-1 pathway

## Abstract

Ulcerative colitis (UC) is an autoimmune inflammatory condition characterized by significant mucosal destruction. Although *Syzygium aromaticum* (Clove oil; CO) oil is well-known for its antioxidant and anti-inflammatory properties, its high volatility, toxicity, and hydrophobicity can compromise its biological efficacy. Therefore, nanoencapsulation is a feasible approach for boosting its therapeutic potential, including stability, bioavailability, and target delivery. Herein, this study was designed to minimize acetic acid-mediated UC using CO alone or encapsulated in nano-vehicles including PCL, CS, and ALG. The developed nano-capsules CO were characterized by Zetasizer, FT-IR, and SEM, and subsequently, the encapsulation efficiency and controlled release profile of CO were determined. Forty adult rats were assigned to five groups (*n* = 8) as follows: the control (CONT) group, which received dimethyl sulfoxide (DMSO) once daily; the UC group, which received rectal acetic acid (AA) instillation on day 8 of the experiment. CO group: rats were treated orally with clove oil (250 mg/kg) dissolved in DMSO once daily. PCL@CS + ALG group; rats were orally treated with nano-vehicle (250 mg/kg). CONPs group: rats received clove oil nanoparticles (PCL@CO(CS + ALG)NPs, 250 mg/kg). All groups received their respective treatments once a day for seven consecutive days, before and after UC induction. An *in* *silico* study revealed the binding affinities of eugenol, the principal bioactive constituent of CO, toward inflammatory molecules at both the mRNA and protein levels. Biologically, the colon outcomes showed that CO, either alone or loaded with nanoparticles (CONPs), significantly decreased MDA and NO levels and elevated antioxidant enzymatic activities (SOD, CAT, , GPx, and GR), with higher GSH levels. Additionally, the treatment of rats with CO or CONPs mitigated colon inflammation by decreasing the MPO activity, TNF-*α*, IFN-*γ*, IL-1*β*, and PGE2 levels, as well as downregulating the expression of NF-*κ*B, IL-6, IL-8, and iNOS genes. Remarkably, CONPs prevented the colon oxidative damage by modulating the mRNA expression of Keap1/Nrf2/HO-1 signaling pathways. Unlike the rats exposed to AA, the treatment with CO and CONPs dramatically restored the mRNA expression of Cdc25c and RNF8 genes. Histologically, the CONPs-treated group showed a clear restoration of colonic tissue architecture toward normal, accompanied by normalization of VEGF and α-SMA immunoexpression patterns. Conclusively, CO, either alone or encapsulated in nanoparticles (CONPs), offers promising therapeutic potential for UC, likely through its anti-inflammatory, antioxidant, and anti-fibrotic effects, as well as superior regulation of angiogenesis compared with pure CO.

## Introduction

Ulcerative colitis (UC) ultimately leads to widespread mucosal disruption and ulceration. It is regarded as a type of crippling inflammatory bowel disease caused by the autoimmune system (Niu et al. 2015). UC can result in significant and sometimes fatal consequences, including colon cancer (Olén et al. 2020), circulatory and respiratory issues (Chu et al. 2017). The elevated production of free radicals and the decreased antioxidant capacity are recognized characteristics of inflammatory bowel disease (IBD) despite the primary etiology of UC remaining unknown (Amirshahrokhi 2019). The pathophysiology of UC is significantly influenced by oxidative stress and excessive inflammation (Tahan et al. 2011). In animal models of colitis, the elevated production of pro-inflammatory cytokines, such as tumor necrosis factor-*α* (TNF-*α*), is crucial (El-Akabawy and El-Sherif 2019). Nuclear transcription factor kappa B (NF-*κ*B) plays a vital role in the induction of UC as it stimulates the production of various inflammatory mediators, including cyclooxygenase-2 (COX-2) (Ahmed et al. 2014). Through the metabolism of arachidonic acid, COX-2 triggers the production and release of prostaglandins in the metabolic pathway of IBD (Le Loupp et al. 2015). Indeed, the massive production of nitrogen and reactive oxygen species (ROS) metabolites impacts the defense mechanism of the intestinal mucosa (Balmus et al. 2016). The damage to the intestinal mucosa may occur through the stimulation of inducible nitric oxide synthase (iNOS) and COX-2, which raises free radicals and suppresses the antioxidant system (Wang and DuBois 2010).

An accessible, reasonably priced, well-researched, and popular experimental paradigm for IBD research is acetic acid (AA)-induced UC (Randhawa et al. 2014). This model resembles the pathophysiology of human IBD and is linked to increased vasopermeability, prolonged neutrophil infiltration, and an elevated inflammatory mediator profile (Thippeswamy et al. 2011). Therefore, AA-induced colitis could serve as a valuable model to evaluate the effectiveness of potential treatments that address the pathophysiology of oxidative stress and inflammation (Low et al. 2013). One significant preventative and therapeutic treatment for UC would be the inhibition of lipid peroxidation and the scavenging of oxygen free radicals (Kassab et al. 2022). Even though there is no known cure for UC, the FDA has approved some medications, including antibiotics, antimicrobials, and anti-inflammatory agents, such as corticosteroids, sulfasalazine, and aminosalicylates (Dubinsky, 2017). However, using these drugs is linked to several adverse side effects, such as nephrotoxicity, hepatotoxicity, headaches, nausea, and epigastric discomfort (Kayal and Shah 2019; Tripathi and Feuerstein 2019). Therefore, there is a growing need to investigate safer and more tolerable alternative medicine alternatives (such as herbal remedies) to treat this illness.

Recent studies have demonstrated that encapsulation-based drug delivery methods can enhance the therapeutic effects of medicines by increasing their stability in vivo, facilitating target accumulation, and improving cellular and tissue absorption (Patil and Deshpande 2018; Zhang et al. 2018). *Syzygium aromaticum* is a tree from the *Myrtaceae* family, originating in Asia and commonly known as clove (Abtahi-Eivari et al. 2021). Clove oil (CO) is an essential oil extracted from the flowers, buds, leaves, and stems of the clove tree. CO is well-known for its antiviral, antifungal, antibacterial, and antioxidant properties (Bakour et al. 2018). These therapeutic properties of *S. aromaticum* extract are due to the presence of eugenol, carvacrol, thymol, and cinnamon aldehyde (Chen et al. 2016), eugenol acetate, and *β*-caryophyllene (Milind and Deepa 2011), which are in charge of the oil's functional properties, including its antioxidant (Gülçin et al. 2012; Nisar et al. 2018), anti-inflammatory (Chaieb et al. 2007), anti-stress (Singh et al. 2009), and antimicrobial capabilities (Cui et al. 2015). The encapsulation of CO or eugenol, through methods, such as microencapsulation, nanoencapsulation, or liposomes, may mitigate the drawbacks of CO or eugenol, including toxicity, low aqueous solubility, poor environmental stability, high pungency, and diminished bioavailability (Cortés-Rojas et al. 2014; Sebaaly et al. 2015; Yadav and Balasubramanian 2016). The protective effects of clove oil nanoemulsion against titanium dioxide nanoparticle toxicity have been previously demonstrated through its enhancement of antioxidant and anti-apoptotic effects, as well as its prevention of genotoxicity (Mohamed et al. 2024). Eugenol makes up 89% of the essential oil found in clove flower buds (Cortés-Rojas et al. 2014). Eugenol has recently been shown to possess numerous biological properties, including anti-proliferative, antioxidant, anticancer, anti-inflammatory, and antimicrobial activities (Al-Trad et al. 2019; Purkait et al. 2020; Fangjun and Zhijia 2018). Polymeric nanoparticles have gained attention recently as a potential novel method for medicine delivery. Among all aliphatic polyester polymers, polycaprolactone (PCL) is the most suitable nano-drug delivery vehicle for various applications due to its distinct and adjustable physicochemical characteristics (Washington et al. 2017; El Yousfi et al. 2023). PCL's low glass transition and melting points, semi-crystallinity, and strong mechanical flexibility enable it to exhibit exceptional drug loading capacity and consistent controlled release characteristics (Sisson et al. 2013; Blázquez-Blázquez et al. 2019). Chitosan (CS) nanoparticles are an example of a functional nano-formulation that stabilizes synthetic medicinal plant essential oils and their active ingredients (Negi and Kesari 2022). For various pharmacological compounds, CS is an economical carrier (Piras et al. 2015). Its cationic charge, inherent antibacterial potential, availability, safety, biodegradability, and biocompatibility are key features that make it an excellent carrier system (Jamil et al. 2016). Another natural biopolymer that can be obtained from brown seaweeds is sodium alginate (ALG), which has potential applications in drug delivery due to its hydrophilicity, biocompatibility, and ease of gelation (Bhattacharyya and Ray 2015).

The desirable features of nanostructures with high surface-area-to-volume ratios boost the CO-releasing capacity of nanoparticles for UC applications. PCL was selected over other polyesters, such as poly(lactic-co-glycolic acid) (PLGA), because it is cost-efficient, lipophilic to enhance passive absorption, and biocompatible (Haas et al. 2005). These attributes, combined with the bioadhesive properties of CS and ALG, are believed to enhance drug absorption through mucosal tissues, such as the bladder wall, and improve cellular interaction. Coated PCLNPs were employed in this study for this purpose, as previous research demonstrated their effectiveness in mucosal drug delivery. Using in vitro characterization methods, including particle size, surface charge, encapsulation efficiency, controlled release, and cellular contact, this study aims to develop and optimize a nanoparticulate carrier based on cationic and anionic polymers for encapsulating CO. According to the existing literature review, no studies have evaluated the efficacy of CO-loaded PCL coated with CS and ALG (CONPs) against AA-induced colitis in rat models. This research was conducted to investigate the potential preventive effects of CONPs (PCL@CO(CS + ALG)NPs) on AA-mediated UC, while also exploring mechanisms related to oxidative stress, inflammation, and histological changes. These results provide new insights for creating safe and effective treatments for UC.

## Materials and methods

### Chemicals

Poly-(vinyl alcohol) (PVA, Mw ~ 13,000–23,000 g/mol, purity 87.0–89.0% hydrolyzed) and PCL (Mw ~ 80,000 g/mol) were purchased from Thermoscientific (UK). CS (Mw ~ 100 KDa), sodium alginate (ALG), and acetic acid (AA, purity 99.7%, Product No. 695092) were obtained from Sigma-Aldrich (St. Louis, MO, USA). Dichloromethane (DCM) HPLC was supplied by Fisher (UK). Sodium chloride (NaCl) was purchased from ADWIC, El Nasr Pharmaceutical Co. Distilled water (D.H_2_O) was used and freshly prepared in the laboratory. Other chemicals were of analytical grade and used directly without further modification.

### Plant material

Air-dried flower buds of cloves (*S. aromaticum*) were collected from a local herbal market in Egypt and recognized by a Plant Taxonomy Consultant at the Egyptian Ministry of Agriculture and Land Reclamation (Mrs. Trease Labib), with identification No. CAIRC-M-174 in the Herbarium of the National Research Centre.

### Essential clove oil preparation and extraction

The European Pharmacopoeia states that a glass Clevenger apparatus, containing 250 g of dried and powdered flower buds in 500 mL of distilled water at a 1:2 ratio, was used to collect the crude CO using the steam distillation process (hydrodistillation). For at least seven hours, boiling water at 100 °C was used to volatilize the essential oils. The separatory funnel will be used to gather and separate the oil following the steam distillation process. Following its recovery, the essential oil was processed with anhydrous sodium sulfate (Na_2_SO_4_) to absorb any remaining water that was present in the oil. Until further investigation, the samples were stored at 4 °C in brown vials.

### Clove oil analysis using gas chromatography–mass spectrometry (GC–MS)

The chemical makeup of the samples was ascertained using a Trace GC-TSQ mass spectrometer (Thermo Scientific, Austin, TX, USA) and a direct capillary column TG–5MS (30 m × 0.25 mm × 0.25 µm film thickness). The column oven's temperature was initially set at 50 °C and then increased by 5 °C every minute to reach 250 °C, where it remained for two minutes. After two minutes, it was finally brought up to 300 °C at a rate of 30 °C per minute. The injector and MS transfer line temperatures were maintained at 270 °C and 260 °C, respectively. Helium was used as the carrier gas at a constant flow rate of 1 mL/min. Following a 4-min solvent delay, diluted samples of 1 µl were automatically injected using the Autosampler AS1300 in the split mode with GC. EI mass spectra were acquired in full scan mode at ionization voltages of 70 eV in the m/z 50–650 range. The ion source's temperature was set at 200 °C. By matching the components' mass spectra to those of the WILEY 09 and NIST 14 mass spectral databases, the components were identified.

### Molecular docking assessment

RNA sequences of NF-*κ*B, IL-6, IL-1*β*, iNOS, Keap1, HMOX1, and Cdc25c were retrieved from the NCBI database; then their three-dimensional (3D) structures were generated using the BIOVIA Discovery Studio Visualizer 2016 software. The conformers of eugenol were generated using the Frog2 (https://bioserv.rpbs.univ-paris-diderot.fr/services/Frog2/) web server. Molecular docking was performed using NLDock_v1.0 operated by Ubuntu 22.04.5 LTS, and their interactions were visualized using BIOVIA Discovery Studio Visualizer 2016 software.

The UniProt (https://www.uniprot.org/) database provided the 3D structures of the proteins NF-*κ*B, IL-6, iNOS, Keap1, and HO-1 in rats, while the PubChem (https://pubchem.ncbi.nlm.nih.gov/) database provided the 3D structure of eugenol. InstaDock software was used for molecular docking (Mohammad et al. 2021), while BIOVIA Discovery Studio Visualizer 2016 was used for visualization.

### Preparation of CONPs

CONPs (PCL@CO(CS + ALG)NPs) were prepared by a simple emulsion-solvent evaporation method. Briefly, the CO (400 mg) and PCL (10% w/v) were dissolved together in 10 mL DCM at room temperature under magnetic stirring for 1 h. PVA (3% w/v) was prepared in 100 mL D.H_2_O under magnetic stirring for 2 h at 80 °C. The PCL@CO solution was introduced dropwise to the PVA solution at room temperature, with stirring for 5 min. Then, the resultant mixture was emulsified with sonication under ice bath for 10 min at the following conditions: temperature 40 °C, amplitude 70%, pulse ON: 5 s., pulse OFF: 15 s. using an ultrasonic probe (SONICS digital Ultrasonic homogenizers Processors, Model VCX 750, USA) to get a more stable and homogenous emulsion. Afterward, the emulsion was stirred for 16 h at room temperature to remove the solvent. The PCL@CONPs were collected at 10,000 rpm for 30 min using a Centrifuge (SIGMA, Germany) and washed three times with deionized H_2_O. The PCL@CONPs were coated with CS and ALG using the layer-by-layer (LBL) method. The PCL@CONPs were dispersed in 0.15 M NaCl and coated with CS (0.1% w/v in 1% AA) at a volume ratio of PCL@CONPs: CS (1:4) under stirring for 15 min. The CS-coated PCL@CONPs were collected at 10,000 rpm for 20 min under centrifugation and washing with D.H_2_O. Subsequently, the CS-coated PCL@CONPs were dispersed in 0.15 M NaCl and coated with ALG (0.1% w/v in D.H_2_O) at a volume ratio of CS-coated PCL@CONPs: ALG (1:4) under stirring for 15 min. The ALG/CS-coated PCL@CONPs [PCL@CO(CS + ALG) NPs] were collected at 10,000 rpm for 20 min under centrifugation and washing with D.H_2_O.

#### UV/Vis spectrophotometric analysis of essential CO

A UV–vis spectrophotometer (Shimadzu spectrophotometer, model UV-900i, Japan) was used to perform spectrophotometric analysis on the vital CO. Using varying concentrations of essential CO-alcoholic solution, the calibration curve was created and shown. The concentrations ranged from 0.06 to 0.6 mg/mL at maximum absorbance (λmax = 282 nm for eugenol). The loading capacity, in vitro controlled-release, and CO encapsulation efficiency were all assessed using the calibration curve.

#### Dynamic light scattering (DLS), polydispersity (PDI), and zeta potential (ZP) analyses of nanoparticles

Using Zetasizer (Zetasizer Advanced series Ultra red label, Malvern Instruments, UK) at a scattering angle of 90°, dynamic light scattering was used to measure the DLS, PDI, and ZP of the produced formulations [PCLNPs, PCL@CONPs, PCL(CS + ALG)NPs, and PCL@CO(CS + ALG)NPs] at 25 ± 2°C. The Brownian motion of the particles was unhindered by diluting 1 mL of the sample with 10 mL of deionized water. Every measurement will be made three times.

#### Fourier-transform infrared spectroscopy analyses of nanoparticles

Fourier-transform infrared spectroscopy (FT-IR) in transmission mode was used to evaluate the chemical composition of the prepared formulations [PCLNPs, PCL@CONPs, PCL(CS + ALG)NPs, and PCL@CO(CS + ALG)NPs] in the 4000–400 cm^−1^ range.

#### Scanning electron microscope analyses of nanoparticles

A scanning electron microscope (SEM) was used to examine the surface morphology of PCL@CO(CS + ALG)NPs (SEM, JOEL 6400 ASID-10, Tokyo, Japan). Gold grids were used to sputter a drop of nanoparticle suspension, which was then examined under a scanning electron microscope.

#### Entrapment efficiency and loading content of formulated PCL@CO(CS + ALG)NPs

The quantity of CO encapsulated inside the PCL and coated with CS/ALG was determined through UV–Visible spectrophotometry (Shimadzu, UV–VIS Spectrophotometer, Model UV-1900i, Japan). Absorbance maximum (λ_max_) of CO in DCM was 282 nm. The colloidal formulations of Nano-Blank [PCL(CS + ALG)NPs] and Nano-CO [PCL@CO(CS + ALG)NPs] (0.3 mL for each) were well-mixed separately in 3 mL DCM and centrifuged at 15,000 rpm for 10 min. By extracting CO in DCM and comparing its absorbance with the calibration curve that was created, the amount of CO loaded was determined. The following formulas were used to determine the loading content (LC) and entrapment efficiency (EE) percentages:

$$\begin{aligned} {\text{EE}}\% \, \% \, & = \, \left( {{\text{Total amount of CO loaded }}/{\text{ Initial amount of CO}}} \right) \, \times { 1}00 \\ {\text{LC}}\% \, & = \, \left( {{\text{Total amount of CO loaded }}/{\text{ Weight of lyophilized nanoemulsion}}} \right) \, \times { 1}00 \\ \end{aligned}$$

#### Controlled essential CO-release from PCL@CO(CS + ALG)NPs

The produced PCL@CO(CS + ALG)NPs were subjected to in vitro drug-release investigations utilizing a dialysis bag membrane with a molecular weight cut-off (MWCO) of 12–14 KDa (Frey Scientific, USA). In summary, 10 mg of dried PCL@CO(CS + ALG)NPs was appropriately suspended in 5 mL of phosphate-buffered saline (PBS, pH 7.4), placed inside the dialysis membrane, clamped on both sides, and submerged in 50 mL of PBS at 37°C with magnetic stirring at 100 rpm. To prevent evaporation, the membrane was then covered with parafilm. Samples were removed from PBS release media at regular intervals (up to about 120 h) and centrifuged for 10 min at 15,000 rpm. The supernatant was then removed and replaced with an equivalent volume of fresh PBS to preserve the initial volume. The supernatant was analyzed spectrophotometrically (UV–Visible spectroscopy) at a wavelength of 282 nm. Nano-Blank PCL (CS + ALG)NPs subjected to the same conditions were maintained as a control. Compared to the nano-vehicle, the release of pure CO (10 mg) was performed too, according to the procedure described above, and PBS was maintained as a control. All measurements were carried out in triplicate. The CO release pattern was obtained by plotting the cumulative amount of CO released at various time intervals versus time using the following formula.

$${\text{Cumulative amount released }} = \frac{{\left( {C \times D} \right)}}{{\left( {\text{Total amount of CO}} \right)}} \times { }100$$

where *C* refers to the concentration of CO in the dissolution medium, and *D* refers to the volume of the dissolution medium.

### Animals

In the animal house of the Department of Clinical Pathology, Faculty of Veterinary Science, Mansoura University, forty male adult albino rats, each weighing 180 ± 10 g, were purchased from an experimental farm in El-Giza. They had unfettered access to water, were boarded in typical circumstances, and were fed appropriately. The animals were kept on a 12-h light/dark cycle in rooms with unlimited access to water. The rats were housed in a laboratory environment with a temperature of 25 ± 2 °C and a humidity of 55 ± 15% for two weeks before the experiment. The animals were not subjected to unnecessary pain or stress, and all animal manipulation was carried out with the highest care and hygiene. The Institutional Animal Ethics Committee at Mansoura University approved the experimental methods following the National Institutes of Health's Guidelines for the Care and Use of Laboratory Animals. The approval number “MU-ACUC (VM.R.25.08.238)”.

### Induction of ulcerative colitis

UC was conducted using acetic acid (AA). On day 8 of the experiment, rats were given moderate anesthesia (pentobarbital, 300 mg/kg) after an overnight fast. Colitis was induced in rats using the technique of Kassab et al. (2022). Briefly, the lower abdomen was palpated to remove the remaining fecal matter after 2 ml of saline were used for colon lavage. Next, using a polyurethane cannula (2 mm in diameter), 2 ml of AA (3%) in normal saline (0.9%) was intrarectally delivered for 30 s. To prevent leakage of acetic acid during instillation, the mice were kept head-down for 2 min. Control animals were given an intracolonial dose of a saline solution (0.9%) in an equivalent volume.

### Treatment protocol and sampling

Forty rats were randomly allocated into five groups (8 animals per group) as follows: Group I (CONT); Rats were treated by oral gavage with 20% dimethyl sulfoxide (DMSO) before and after UC induction. Group II (UC group): Rats received rectal AA instillation at day 8 of the experiment. Group III (CO); Rats were treated with CO (250 mg/kg) dissolved in 20% DMSO orally once daily , according to Santin et al. (2011). Group IV (PCL@CS + ALG); Rats were orally treated with nano-vehicle PCL@(CS + ALG)NPs (250 mg/kg) . Group V (CONPs); Rats received PCL@CO(CS + ALG) NPs orally at a dose of 250 mg/kg . Before and after UC was induced, all groups received the therapy once a day for seven consecutive days.

In our study, the selected dose of clove oil (CO) nanoemulsion (250 mg/kg) was based on previous toxicity evaluations and OECD guidelines. The maximum tolerated dose (MTD) and half lethal dose (LD_50_) of CO nanoemulsion were above 2000 mg/kg (Mohamed et al. 2024). Their study further demonstrated that oral administration of CO nanoemulsion at 100 mg/kg (5% of the LD_50_) produced no signs of toxicity or mortality during a 14-day monitoring period. In line with these findings, we conducted a preliminary acute toxicity test in our laboratory following the OECD/OCDE primary protocol, where rats received oral doses up to 5 g/kg. All animals remained healthy, with no observable behavioral changes, toxicity signs, or mortality during the observation period. Accordingly, the LD_50_ of CO nanoemulsion was considered greater than 5 g/kg. Based on these results, the therapeutic dose for the present study was calculated as 5% of the safety dose, corresponding to 250 mg/kg body weight.

 After completing the respective treatments, all rats were sacrificed the following day using sodium pentobarbital (Somnopentyl, 50 mg/kg, i.p.; Kyoritu Pharmaceutical Co., Ltd., Tokyo, Japan). A Samsung camera (model WB30F, Japan) was used to take, weigh, clean, and photograph the colon samples after the animals were euthanized by cervical dislocation. To perform histological and immunohistochemical studies, a centimeter from the resected colon was preserved in 10% formalin. Utilizing a polytron homogenizer (Tri-R Instruments, Inc., Rockville Centre, NY; Tri-R Stir-R homogenizer). To assess the mRNA levels of the chosen genes, total RNA was extracted from a different section of the tissue. The remainder of the colon was homogenized in ice-cold phosphate buffer (pH 7.4) at a weight-to-volume ratio of approximately 1:5. The homogenate was then centrifuged at 5000 rpm for 15 min, and the supernatant was divided into aliquots. Aliquots were prepared and stored for further analysis at -80 °C.

### Colon oxidative/antioxidant status

In colon tissues, the lipid peroxidation marker malondialdehyde (MDA), nitric oxide (NO), total superoxide dismutase (T-SOD), catalase, glutathione (GSH), glutathione peroxidase (GPx), and glutathione reductase (GR) were colorimetrically determined using the methodology described by Ohkawa et al. (1979), Green et al. (1982), Nishikimi et al. (1972), Aebi (1984), Ellman (1959), Paglia et al. (1967), and Smith et al. (1988), respectively. Afterward, Bradford (1976) techniques were used to assess the protein content in the tissue homogenates in order to compute oxidative/antioxidant indicators per milligram of protein.

### Colon inflammatory mediators

Using specialized ELISA kits for rats purchased from MyBioSource company (California, USA), the colon levels of prostaglandin E2 (PGE2, catalog No.; MBS730592), TNF-*α* (catalog No.; MBS2507393), interferon-gamma (IFN-*γ*, catalog No.; MBS2500392), interleukin 1 beta (IL-1*β*, catalog No.; MBS825017), and interleukin 10 (IL-10, catalog No.; MBS2707969) were assessed. Additionally, myeloperoxidase (MPO) activity was measured spectrophotometrically as an indicator of neutrophil infiltration using the method described by Bradley et al. (1982).

### RNA extraction, cDNA synthesis, and quantitative real-time PCR

Following the manufacturer's instructions, total RNA was isolated from the colon using the Direct-zolTM RNA MiniPrep Trizol reagent (catalog No. R2050). A Nanodrop (UV–Vis spectrophotometer Q5000/USA) was used to test the quantity and purity, and gel electrophoresis was used to assess the integrity. Following the manufacturer's instructions, cDNA was synthesized using the SensiFast cDNA Synthesis Kit from Bioline (catalog number Bio-65053). The reaction mixture consisted of 1 μg of total RNA, 4 μl of 5 × Trans Amp buffer, 1 μl of reverse transcriptase, and 20 μl of DNase-free water, totaling 26 μl. The following program was run after the final reaction mixture was placed in a heat cycler: primer annealing for 10 min at 25 °C, reverse transcription for 15 min at 42 °C, and inactivation for 5 min at 85 °C.

SYBR Green PCR Master Mix (2 × SensiFastTM SYBR, Bioline, catalog No.; Bio-98002) was utilized to measure the mRNA levels of inflammatory (NF-*κ*B, IL-6, IL-8, and iNOS), antioxidant (Nrf2, Keap1, and HO-1), and DNA damage (cell division cycle protein 25c (Cdc25c) and ring finger protein 8 (RNF8)) indicators in the colon. Table 1 displays the size of each amplified PCR product along with the primer sequences. As an internal control, the housekeeping gene *β*-actin was employed. The reaction mixture, which contained 10 μl of 2 × SensiFast SYBR, 3 μl of cDNA, 5.4 µl of H_2_O (dH_2_O), and 0.8 µl of each primer, was conducted in a total volume of 20 μl. The PCR cycling conditions were as follows: 95 °C for 4 min, followed by 40 cycles of 94 °C for 15 s, annealing temperatures as shown in Table 1 for 30 s, and an extension temperature of 72 °C for 20 s. A melting curve analysis was performed after the amplification phase to verify the specificity of the PCR product. The 2^−ΔΔCt^ method is used to calculate the relative expression of each gene in each sample compared to the control gene and the *β*-actin gene (Pfaffl 2001).

**Table 1:** Primers used for real-time PCR amplifications

Genes	GenBank accession numbers	Oligonucleotide sequences	Annealing temperature (°C)	Sizes (bp)
*NF-ĸβ*	AF079314.2	f5,—TGGACGATCTGTTTCCCCTC -3, r5,—CCCTCGCACTTGTAACGGAA-3,	58	118
*IL-6*	NM_012589.2	f5,—AGCGATGATGCACTGTCAGA-3, r5,—GGAACTCCAGAAGACCAGAGC-3,	60	127
*IL-8*	NM_031512.2	f5,—GACTTCACCATGGAACCCGT -3, r5,—GGAGACTGCCCATTCTCGAC -3,	58	104
*iNOS*	NM_012611.3	f5,—TGGGTGAAAGCGGTGTTCTT -3, r5,—TAGCGCTTCCGACTTCCTTG -3,	60	108
*Nrf2*	NM_031144.3	f5,—GTCCACCCGCGAGTACAACCT-3, r5,—GGAGCCGTTGTCGACGACGA-3,	60	119
*Keap1*	NM_057152.2	f5,—TCCTCAGAGGGCAGTGGAAT-3, r5,—TATGTGTCCCACAAGGGAGC-3,	60	159
*HO-1*	NM_012580.2	f5,—GCCTGGTTCAAGATACTACCTCT-3, r5,—CTGAGTGTGAGGACCCATCG-3,	58	103
*Cdc25c*	NM_001401323.1	f5,—TCCTTGTCATTCGGTGGAGTC-3, r5,—ACCGAGCGAGGTGGAGCTA-3,	60	209
*RNF8*	NM_001025727.1	f5,—TGAGTCCAGAACCAACTCGC-3, r5,—TCTCTTTGCTCTTCGTTCCCT-3,	58	118
*β-Actin*	NM_031144.3	f5,—GGCATGTGCAAGGCCGGCTT -3, r5,—TAGGAGTCCTTCTGACCCATA -3^,^	58	116

### Weight/length ratio, macroscopic and microscopic evaluations of the colon

The colon's weight-to-length ratio was measured after it was removed from the cecum to the anus (Kassab et al. 2022). To evaluate the extent of colonic inflammation macroscopically and microscopically, colons were collected from fasted, scarified rats (7 cm above the anal margin), washed with phosphate-buffered saline. Macroscopic damage scores, assessing the presence or absence of ulcers and the area of inflammation, were evaluated according to Wallace and Keenan (1990). The macroscopic damage score was graded from 0 (no ulcer, no inflammation) to 5 ulcerations extending more than 2 cm. For microscopic analyses, the distal colon (the most affected area) was transversely cut and fixed in 10% neutral buffered formalin for 48 h. The fixed samples were dehydrated using an ascending series of ethyl alcohol, cleared with xylene, and embedded in liquid paraffin wax for blocking. Five μm sections were cut by the rotatory microtome and mounted on coated glass slides for H&E (to assess the colitis score) and alcian blue (for goblet cells staining) stains (Bancroft et al. 2013) or mounted on positive glass slides for immunohistochemical staining for *α* smooth muscle actin (*α*-SMA) and vascular endothelial growth factor (VEGF) according to Petrosyan et al. (2002).

### Immunohistochemical expressions of VEGF and *α*-SMA

Briefly, the mounted sections on positive glass slides were deparaffinized, rehydrated, and then treated with 3% H_2_O_2_ for 5 min at room temperature to block endogenous peroxidase activity. The slides were washed with PBS and blocked with 10% normal goat serum then incubated overnight in the dark humified chamber at 4.0c with primary *α*-SMA (rabbit, polyclonal, 1:200 dilution, CAT NO. 55,135–1-AP) or primary VEGF (rabbit, polyclonal, 1:150 dilution, CAT NO. AB1876-I), then washed with PBS and incubated with biotinylated secondary antibody (polyclonal anti-rabbit, 1:200 dilution, CAT NO. A0545) for 1 h at room temperature. The sections were incubated for 10 min with diaminobenzidine and counterstained with hematoxylin. The histological score (HS) of the colitis was evaluated according to Li et al. (2013) and Engel et al. (2008), including inflammation extent (0–3), damage in crypt architecture (0–2), edema/hyperemia in the submucosa (0–3), and mononuclear inflammatory cells infiltration (0–3) scores. The percentage of mean area of alcian blue-positive reaction for the goblet cells, the immune density % of *α*-SMA, and VEGF expression were also analyzed. For the histomorphometric analyses, five non-overlapping sections (× 40) for each rat (three rats/group) were randomly selected and analyzed using the ImageJ program version 1.36 (NIH, USA).

### Statistical analysis

The Statistical Package for the Social Sciences (SPSS, version 17) was used to analyze the data and present the results as means with standard deviation (SD). The data analysis program GraphPad Prism (version 6.1; GraphPad Software, Inc., San Diego, CA, USA) was used to construct the histogram figures. The variance across groups was assessed using a one-way analysis of variance (ANOVA). An acceptable level of significance was defined as a P value of less than 0.05. Furthermore, a clustering heatmap was created using RStudio software, version 2023.12.0 “Ocean Storm” Release (33206f75, 2023-12-17) for Windows, under R version 4.0.2, to highlight the most critical variables in the current study and further explain the interrelationships among all the variables.

## Results

### GC–MS analysis of clove oil

**Fig. 1:**
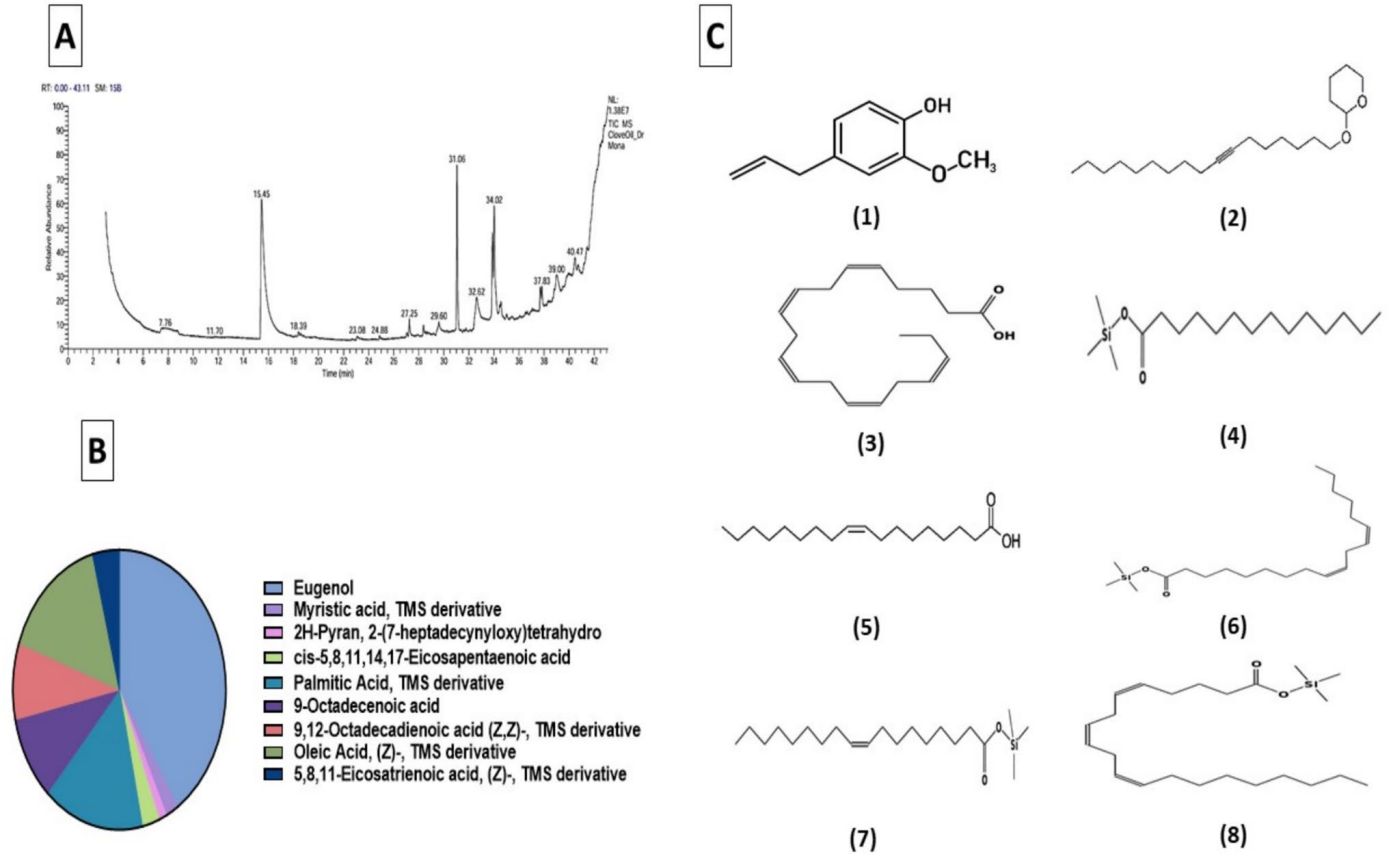
**A** Profile of GC–MS analysis for clove oil. **B** Relative abundance of phenolic acids and flavonoids in clove oil. The chemical structure of the main identified components (1) eugenol, (2) 2H-Pyran, 2-(7-heptadecynyloxy)tetrahydro, (3) cis-5,8,11,14,17-Eicosapentaenoic acid, (4) palmitic Acid, TMS derivative, (5) 9-Octadecenoic acid, (6) 9,12-Octadecadienoic acid (Z,Z)-, TMS derivative, (7) oleic Acid, (Z)-, TMS derivative, and (8) 5,8,11-Eicosatrienoic acid, (Z)-, TMS derivative

The main compound of clove oil was eugenol (40.76%) (Fig. 1A, B). Additionally, the other main compounds were myristic acid, TMS derivative (1.95%), 2H-Pyran, 2-(7-heptadecynyloxy)tetrahydro (1.19%), cis-5,8,11,14,17-Eicosapentaenoic acid (2.62%), palmitic acid, TMS derivative (15.26%), 9-Octadecenoic acid (9.82%), 9,12-Octadecadienoic acid (Z, Z)-, TMS derivative (8.81%), oleic acid, (Z)-, TMS derivative (15.54%), and 5,8,11-Eicosatrienoic acid, (Z)-, TMS derivative (4.05%) (Fig. 1).

### Molecular docking interactions

**Fig. 2:**
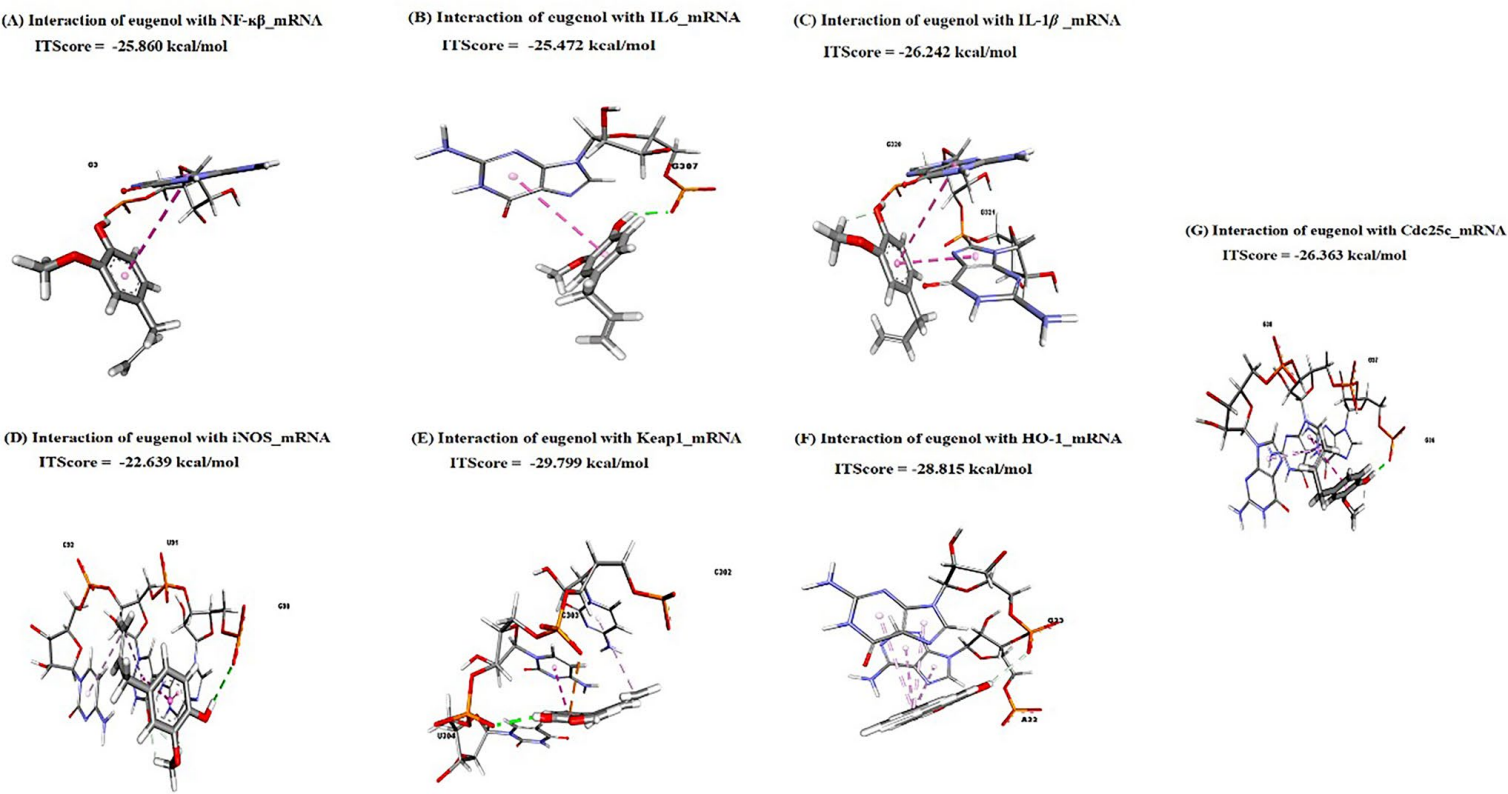
Molecular docking interaction of eugenol with **A** nuclear factor kappa B (NF-*κ*B), **B** interleukin-6 (IL-6), **C** interleukin-1 beta (IL-1*β*), **D** inducible nitric oxide synthase (iNOS), **E** Keap1, kelch-like ECH-associated protein 1 (Keap1), **F** heme oxygenase 1 (HO-1), and (**G**) cell division cycle protein 25c (Cdc25c) mRNAs

Data represented in Table 2 and Fig. 2A–G revealed the molecular interactions of eugenol with NF**-***κ*B, IL6, IL1*β*, iNOS, Keap1, HO-1, and Cdc25c mRNAs, where eugenol interacted with them by scores of -25.860, -25.472, -26.242, -22.639, -29.799, -28.815, and -26.363 kcal/mol, respectively. Eugenol interacted with G3 and G307 nucleotides in *NF-κB* and *IL-6*, respectively, by one hydrogen bond and one hydrophobic interaction. In comparison, bound with G320 (hydrogen bond) and G321 (hydrophobic interaction) nucleotides in *IL-1β*. In the ***iNOS*** mRNA, eugenol interacted with G90 (hydrogen bond), U91 (hydrogen bond), and C92 (hydrophobic interaction) nucleotides. Additionally, it is bound to C302 (hydrophobic interaction), C303 (hydrophobic interaction and charge), and U304 (hydrogen bond) nucleotides in *Keap1* mRNA. Furthermore, eugenol interacted with *HO-1* mRNA in A22 (hydrophobic interaction) and G23 (hydrogen bond and hydrophobic interaction) nucleotides. Finally, eugenol interacted with G36 (hydrogen bond), G37 (hydrophobic interaction), and G38 (hydrophobic interaction) nucleotides in *Cdc25c* mRNA.

**Table 2:** Molecular interactions of eugenol against nuclear factor kappa B (NF-*κ*B), interleukin-6 (IL-6), interleukin-1 beta (IL-1*β*), inducible nitric oxide synthase (iNOS), Keap1, kelch-like ECH-associated protein 1 (Keap1), heme oxygenase 1 (HO-1), and cell division cycle protein 25c (Cdc25c) mRNAs

Targets	Residues	Hydrogen bond	Charge	Hydrophobic interaction
NF-κB	G3	1	0	1
IL-6	G307	1	0	1
IL-1β	G320	1	0	1
	G321	0	0	1
iNOS	G90	1	0	1
	U91	1	0	1
	C92	0	0	1
Keap1	C302	0	0	1
	C303	0	1	1
	U304	1	0	0
HO-1	A22	0	0	1
	G23	1	0	1
Cdc25c	G36	1	0	0
	G37	0	0	1
	G38	0	0	1

**Fig. 3:**
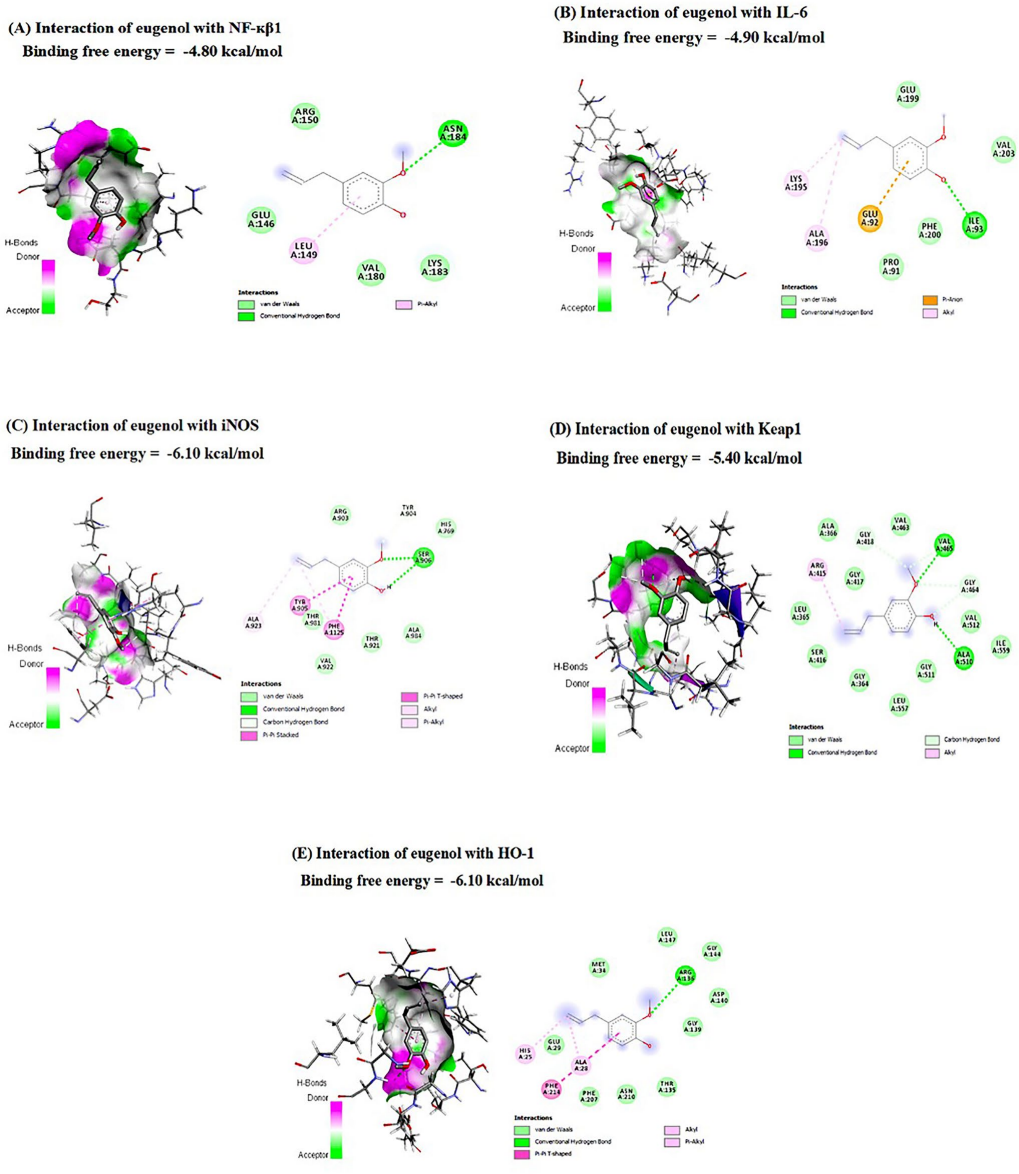
Molecular docking interaction of eugenol with **A** nuclear factor Kappa B (NF-*κ*B), **B** interleukin-6 (IL-6), **C** inducible nitric oxide synthase (iNOS), **D** kelch-like ECH-associated protein 1 (Keap1), and (**E**) heme oxygenase 1 (HO-1) proteins

At the protein level, eugenol interacted with the binding sites of NF-*κ*B, IL-6, iNOS, Keap1, and HO-1, with binding free energies of -4.80, -4.90, -6.10, -5.40, and -6.10 kcal/mol, respectively (Table 3 and Fig. 3A–E). Eugenol interacted with the NF-*κ*B-binding site through hydrophobic interactions (at the LEU149 residue) and hydrogen bonding (at the ASN184 residue). Additionally, it is bound by LEU83 (hydrophobic interaction), LEU90 (hydrophobic interaction), PRO91 (hydrogen bond), and ALA196 (hydrophobic interaction) residues in the binding site of IL-6. By hydrogen bonds (TYR904 and SER906 residues) and hydrophobic interactions (TYR905, ALA923, and PHE1125 residues), eugenol interacted with iNOS’s binding site. In addition, eugenol interacted with ARG415 (hydrophobic interaction), GLY418 (hydrogen bond), GLY464 (hydrogen bond), VAL465 (hydrogen bond), and ALA510 (hydrogen bond) in the binding site of Keap1. Also, it is bound with hydrogen bonds (ARG136 residue) and hydrophobic interactions (HIS25, ALA28, and PHE214 residues) in the binding site of HO-1.

**Table 3:** Molecular interactions of eugenol against nuclear factor kappa B (NF-*κ*B), interleukin-6 (IL-6), inducible nitric oxide synthase (iNOS), Keap1, kelch-like ECH-associated protein 1 (Keap1), and heme oxygenase 1 (HO-1) proteins

Targets	Residues	Hydrogen bond	Charge	Hydrophobic interaction
NF-*κ*B	LEU149	0	0	1
	ASN184	1	0	0
IL-6	LEU83	0	0	1
	LEU90	0	0	1
	PRO91	1	0	0
	ALA196	0	0	1
iNOS	TYR904	1	0	0
	TYR905	0	0	1
	SER906	1	0	0
	ALA923	0	0	1
	PHE1125	0	0	1
Keap1	ARG415	0	0	1
	GLY418	1	0	0
	GLY464	1	0	0
	VAL465	1	0	0
	ALA510	1	0	0
HO-1	HIS25	0	0	1
	ALA28	0	0	1
	ARG136	1	0	0
	PHE214	0	0	1

### UV/Vis spectrophotometric analyses of essential CO

**Fig. 4:**
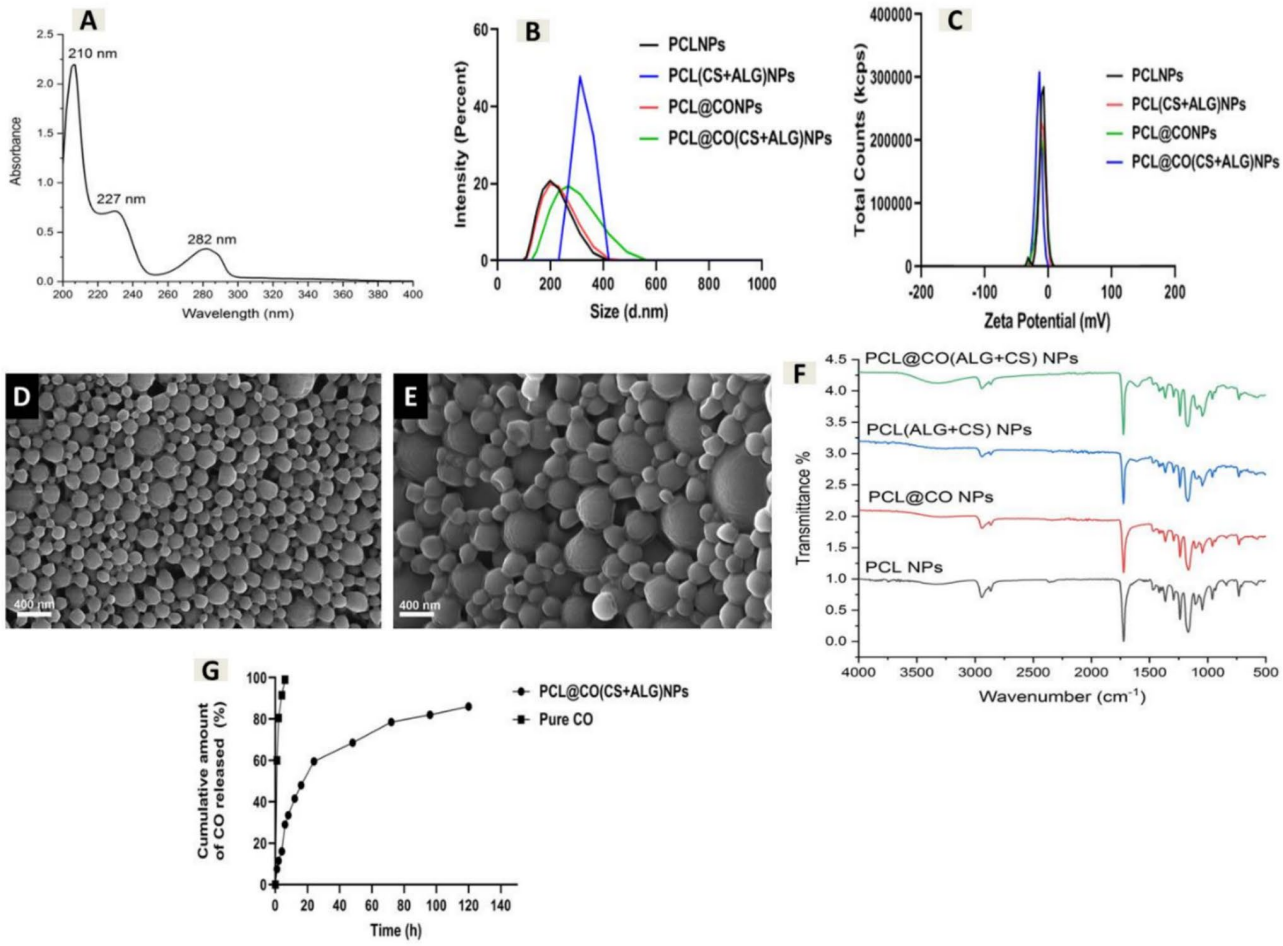
Characterizations of the prepared particles. **A** UV–Vis absorption spectra of essential CO, **B** DLS measurements of nano-blank and nano-CO formulations, **C** zeta potential (ζ) measurements of nano-blank and nano-CO formulations, **D** SEM image of formulated PCL@CO NPs, **E** SEM image of formulated PCL@CO (ALG + CS) NPs, **F** FT-IR spectra of nano-blank and nano-CO formulations, and **G** the in vitro release profile of essential CO from PCL@CO(CS + ALG) NPs compared to pristine CO

In addition to the GC–MS profile, the absorption spectra of vital CO were evaluated to show the absorption maximum (λmax) of the active components present in CO. The spectrophotometric determination of the absorption spectra of essential CO revealed that the absorption maxima (λmax) for geraniol, linalool, and eugenol were at 210 nm, 227 nm, and 282 nm, respectively (Shimadzu spectrophotometer, model UV-900i, Japan). Various concentrations of essential CO-alcoholic solutions, ranging from 0.06 to 0.6 mg/mL, were used to prepare the calibration curve. The concentration was plotted on the x-axis, and the corresponding first derivative value was plotted on the y-axis (y = 0.237394 x + 0.00442109, R2 = 0.9996) (Fig. 4A).

### Dynamic light scattering (DLS), polydispersity (PDI), and zeta potential (ZP)

The DLS, PDI, and ZP for the created NP formulations are displayed in Fig. 4B, C, which also reveals the alteration features during the creation of nanoparticles and nanoencapsulation. PCLNPs, PCL(CS + ALG)NPs, PCL@CONPs, and PCL@CO(CS + ALG)NPs formulations had DLSs of 194.1 ± 0.90, 451.6 ± 1.8, 202.5 ± 0.76, and 261.1 ± 0.53 nm, respectively, according to the results. PCL NPs displayed a lower particle size because of the PCL chain's increased flexibility. The successful trapping of ALG and CS on the surface of nanoparticles and the CO in the core, along with polymer PCL, was confirmed by the increased particle size in the coated samples containing ALG and CS, as well as polymer PCL. All generated NP formulations exhibit a limited size distribution, as indicated by the PDI value, which is less than 1 and falls within the range of 0.05 to 0.12. PCLNPs, PCL(CS + ALG)NPs, PCL@CONPs, and PCL@CO(CS + ALG)NPs formulations had zeta potential values of − 9.85, − 8.87, − 9.58, and − 16.67 mV, respectively.

### Scanning electron microscope

The surface morphology of CONPs (PCL@CO(CS + ALG)NPs) formulations, which display the nanoencapsulation characteristics of essential CO, is clarified by the SEM images shown in Fig. 4D, E. These pictures show the nanoparticle formulations' spherical, regular, and smooth surfaces both before and after drug encapsulation. The enclosed nanoparticles exhibited a homogeneous size distribution as evidenced by the nano-formulations appearing somewhat larger than the particle size determined by the light scattering approach.

### Fourier-transform infrared spectroscopy

To understand the potential interactions between the various components of the created bionanocomposite, FT-IR analysis was conducted. The generated formulations' chemical composition (PCLNPs, PCL(CS + ALG)NPs, PCL@CO(CS + ALG)NPs, and PCL@CONPs) and the presence of CO in the nano-formulations are shown in Fig. 4F. The modulation in the C–H asymmetric stretching was revealed by a modest shift in the band and variations in the peak strength, which also validated the interaction between the matrix's constituent components. All the produced bionanocomposites had typical PCL peaks at 2947.396 and 2867.460 cm^−1^, which corresponded to the symmetric and asymmetric C–H stretching bonds (aromatic ring and aliphatic chain), respectively. However, CH_2_ bending was responsible for the peak at 1367.116 cm^−1^ in the spectrum. Additional peaks corresponding to carbonyl (C=O) stretching and the asymmetric and symmetric stretching of C–O–C bonds were found at 1725.805, 1244.137, and 1166.250 cm^−1^, respectively. The FT-IR spectra revealed peaks at 2742.660 cm^−1^ during coating with ALG and CS, showing both intramolecular and intermolecular hydrogen bond stretching band overlap in the O–H and N–H directions. As a result, there may be some interactions between the O–H groups in CO, CS, and ALG that cause minor changes in the O–H stretching area. There was a peak for C = O stretching of ALG at 1604.875 cm^−1^. With the addition of CO, the C–O groups in these materials may be responsible for the peak at 1046 cm^−1^. Other small peaks that were noticed, however, might have been caused by interactions between these substances, including esterification or hydrogen bonds.

### Entrapment efficiency and loading content of formulated PCL@CO(CS + ALG)NPs

To ascertain the loading capacity and encapsulation efficiency of the prepared PCL@CO(CS + ALG)NPs, the standard curve of the CO was displayed. Desired activity against UC requires a desirable loading content of CO and a desirable entrapment efficiency. The biological characteristics of vital CO may be compromised because of its high volatility, toxicity, and hydrophobic properties. Thus, in this work, the use of CS and ALG for CO nanoencapsulation was a viable method for increasing the therapeutic potential by optimizing their target delivery, stability, and bioavailability. Consequently, the current investigation determined the percentage of EE and the percentage of LC of PCL@CO(CS + ALG)NPs. It was discovered that the characteristics of the polymers primarily determined the EE% and LC% of CONPs (PCL@CO(CS + ALG)NPs). The findings showed that the percentage of LC was 20.39 percent, and the percentage of EE was 91.85%. These results may be explained by the high hydrophobic nature of PCL as well as the effectiveness of CS and ALG in reducing CO leakage from nanoparticles. Furthermore, the high %LC indicates that CO was taken up by the nanoparticle surface following interaction between negatively charged PCL containing CO and positively charged CS and negatively charged ALG.

### Controlled essential CO release

The in vitro release of essential CO from PCL@CO(CS + ALG)NPs in comparison to pristine CO was carried out in phosphate buffer solution (pH 7.4) as illustrated in the section "Entrapment efficiency and loading content," and PCL@CO(CS + ALG)NPs were the best encapsulation system chosen based on the encapsulation efficiency. The findings showed that pure vital CO had a high burst of around 60% in the first hour and a rapid release rate of almost 100% after six hours. However, an initial burst release magnitude of 16% was observed in the first 4 h, followed by a sustained progressive release of 29%, 33.5%, 41.5%, and 48%, which persisted for approximately 120 h. This biphasic pattern was evident in the sustained release of essential CO from PCL@CO(CS + ALG)NPs (Fig. 4G).

### CONPs attenuated oxidative stress in acetic acid-induced ulcerative colitis.

**Fig. 5:**
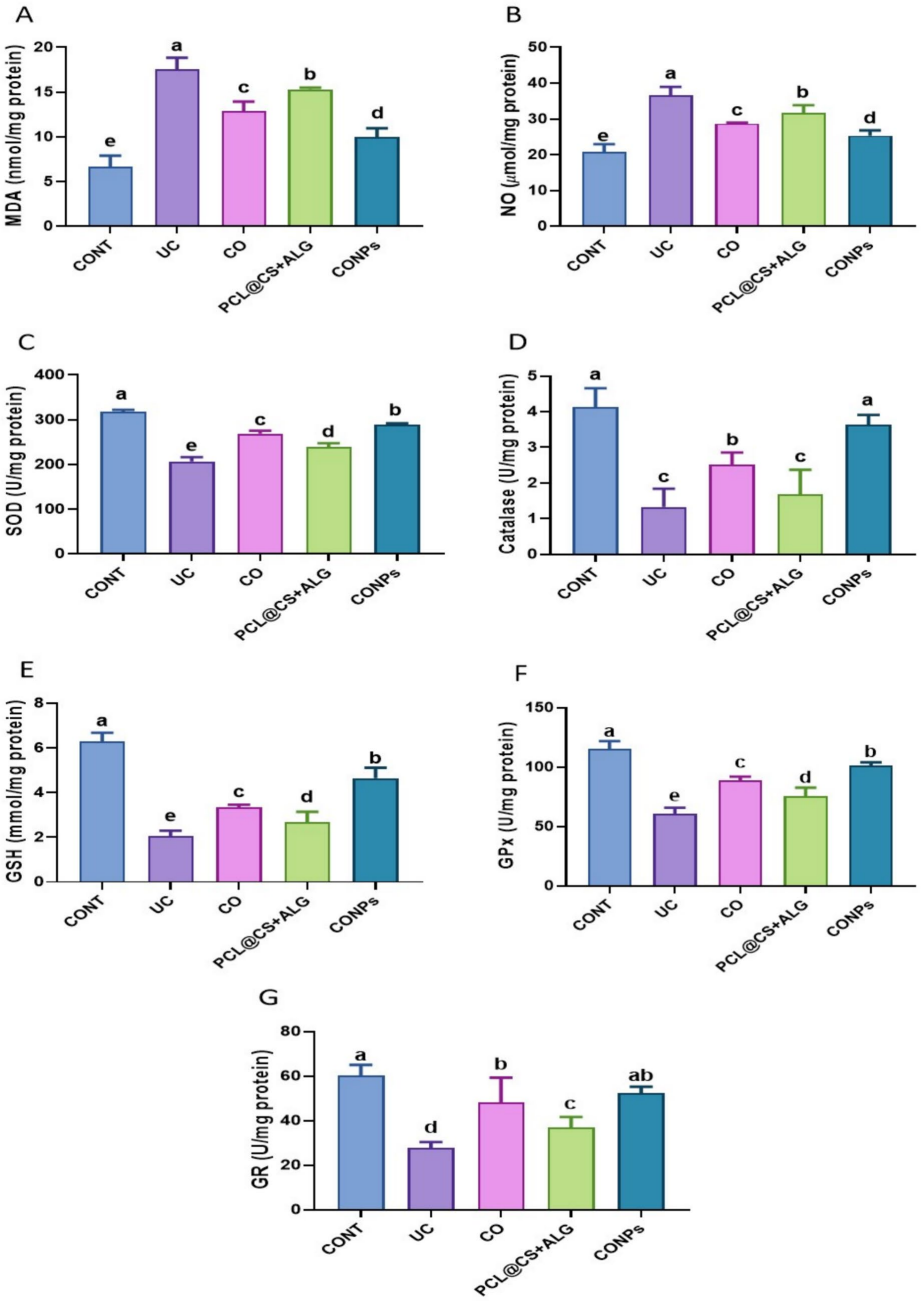
Attenuating effects of *Syzygium aromaticum* (Clove) oil nanoparticles (PCL@CO(CS + ALG)NPs; CONPs) on colon oxidative stress markers; malondialdehyde (MDA), nitric oxide (NO); as well as antioxidant molecules; superoxide dismutase (SOD), catalase, reduced glutathione (GSH), glutathione peroxidase (GPx), and glutathione reductase (GR) upon exposure to acetic acid-mediated ulcerative colitis (UC) in rats. Data were expressed as mean ± SEM (n = 5/ group). Letters (a, b, c, d) refer to the significant differences when comparing rat groups, at *p values* < 0.05

Rats exposed to acetic acid (UC group) had higher colon levels of lipid peroxides (MDA and NO) than the control group (*p* < 0.05) as indicated by oxidative stress markers (Fig. 5). Thankfully, CO, PCL@CS + ALG, and CONPs supplementation restored the colon's MDA and NO levels compared to the intoxicated, untreated group (*p* < 0.05).

Figure 5 demonstrates that, in contrast to the control group (*p* < 0.05), acetic acid poisoning is associated with reduced levels of antioxidant enzyme molecules (T-SOD, catalase, GSH, GPx, and GR) in colon tissue and contrast to the UC group, the co-administration of CO, PCL@CS + ALG, and CONPs improved the reduced antioxidant enzyme activity (*p* < 0.05). Colon catalase and GR activity returned to normal after co-treatment with CONPs, comparable to the control rats' levels (*p* < 0.05) (Fig. 5). Remarkably, the group treated with CONPs showed increased levels of antioxidant molecules and lower levels of oxidative biomarkers.

### CONPs alleviated inflammatory mediators in acetic acid-induced ulcerative colitis

**Fig. 6:**
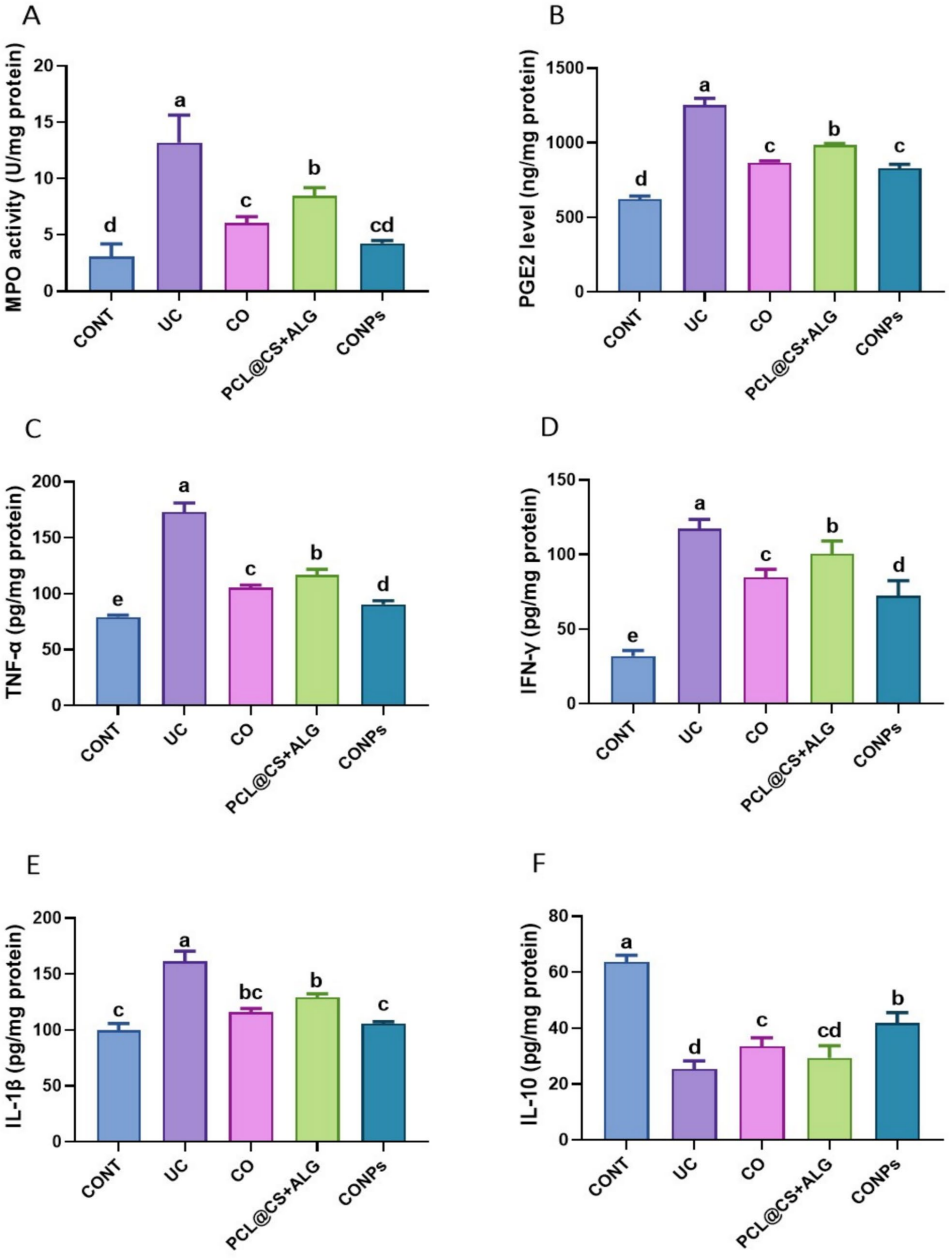
Attenuating effects of *Syzygium aromaticum* (Clove) oil nanoparticles (PCL@CO(CS + ALG)NPs; CONPs) on colon myeloperoxidase (MPO) activity, prostaglandin E2 (PGE2), tumor necrosis factor-*α *(TNF-*α*), interferon-gamma (IFN-*γ*), interleukin 1 beta (IL-1*β*), and interleukin 10 (IL-10) upon exposure to acetic acid-mediated ulcerative colitis (UC) in rats. Data were expressed as mean ± SEM (n = 5/ group). Letters (a, b, c, d) refer to the significant differences when comparing rat groups, at *p values* < 0.05

Acetic acid exposure caused inflammation in the colon as evidenced by a significant decrease (*p* < 0.05) in IL-10 levels and a significant increase (*p* < 0.05) in inflammatory mediator levels (TNF-*α*, IFN-*γ*, and IL-1*β*) compared to the control group. Additionally, when compared to the control group, the intrarectal injection of AcOH significantly (*p* < 0.05) raised the levels of PGE2 and MPO activity. On the other hand, after UC was induced, there was a significant decrease in inflammatory markers, particularly in the CONPs-treated group (*p* < 0.05), suggesting that treatment with CO, PCL@CS + ALG, and CONPs reduced colon inflammation. This indicates that CONPs exhibit anti-inflammatory properties against colon inflammation induced by acetic acid injection (Fig. 6).

### CONPs restored the gene expression in acetic acid-induced ulcerative colitis

**Fig. 7:**
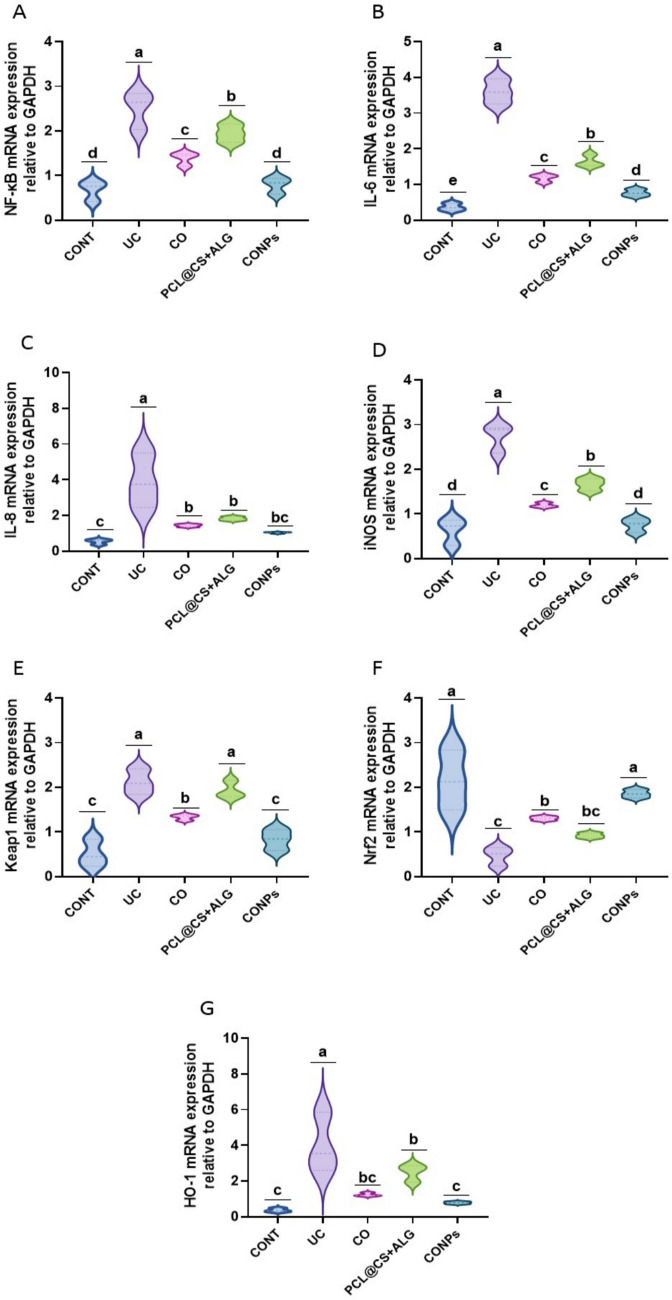
Attenuating effects of *Syzygium aromaticum* (Clove) oil nanoparticles (PCL@CO(CS + ALG)NPs; CONPs) on colon nuclear factor Kappa B (NF-*κ*B), interleukin 6 (IL-6), interleukin 8 (IL-8), inducible nitric oxide synthase (iNOS), nuclear factor erythroid 2–related factor 2 (Nrf2), kelch-like ECH-associated protein 1 (Keap1) and heme oxygenase 1 (HO-1) upon exposure to acetic acid-mediated ulcerative colitis (UC) in rats. Data were expressed as mean ± SEM (*n* = 3/ group). Letters (a, b, c, d) refer to the significant differences when comparing rat groups, at *p values* < 0.05

**Fig. 8:**
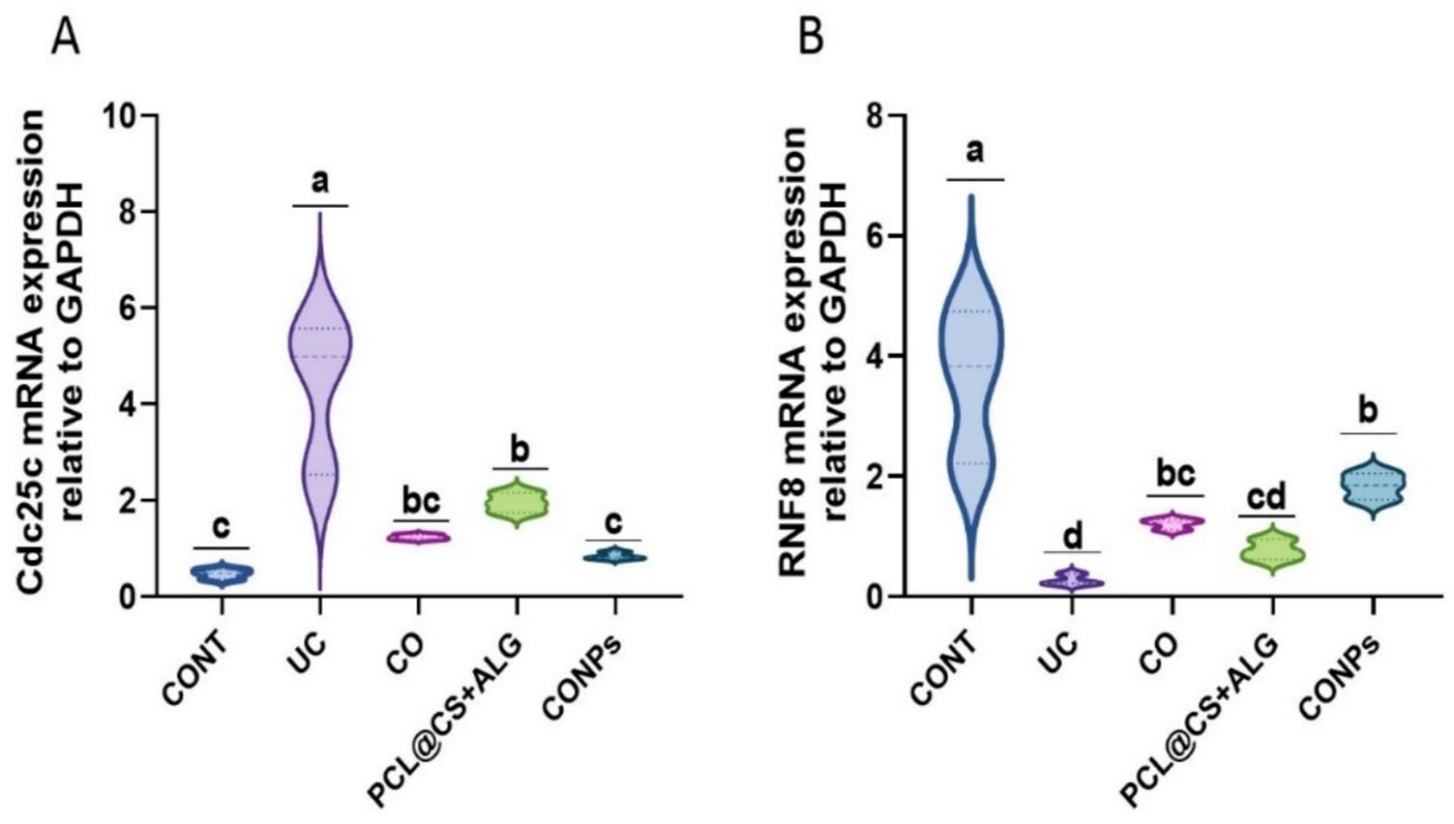
Attenuating effects of *Syzygium aromaticum* (Clove) oil nanoparticles (PCL@CO(CS + ALG)NPs; CONPs) on colon expression of cell division cycle 25C (Cdc25c) and E3 ubiquitin-protein ligase (RNF8) genes upon exposure to acetic acid-mediated ulcerative colitis (UC) in rats. Data were expressed as mean ± SEM (n = 3/ group). Letters (a, b, c, d) refer to the significant differences when comparing rat groups, at *p values* < 0.05

In general, acetic acid may alter the expression of indicators for inflammation, antioxidants, and DNA damage in the colon (Figs. 7 and 8). Acetic acid markedly up-regulated (*p* < 0.05) NF-*κ*B, IL-6, IL-8, iNOS, Keap1, HO-1, and Cdc25c relative to the control group. However, Nrf2 and RNF8 mRNA expressions were reduced (*p* < 0.05). When compared to rats treated with acetic acid, treatment with clove oil, PCL@CS + ALG, and CONPs dramatically reduced (*p* < 0.05) the mRNA expression of NF-*κ*B, IL-6, IL-8, iNOS, Keap1, HO-1, and Cdc25c. On the other hand, the Nrf2 and RNF8 genes produced the opposite pattern (*p* < 0.05). The rats treated with CONPs showed the greatest ameliorative impact.

### CONPs mitigated the histological alteration in the colon upon exposure to acetic acid

#### Weight /length ratio and macroscopic ulcerative colitis scores

**Fig. 9:**
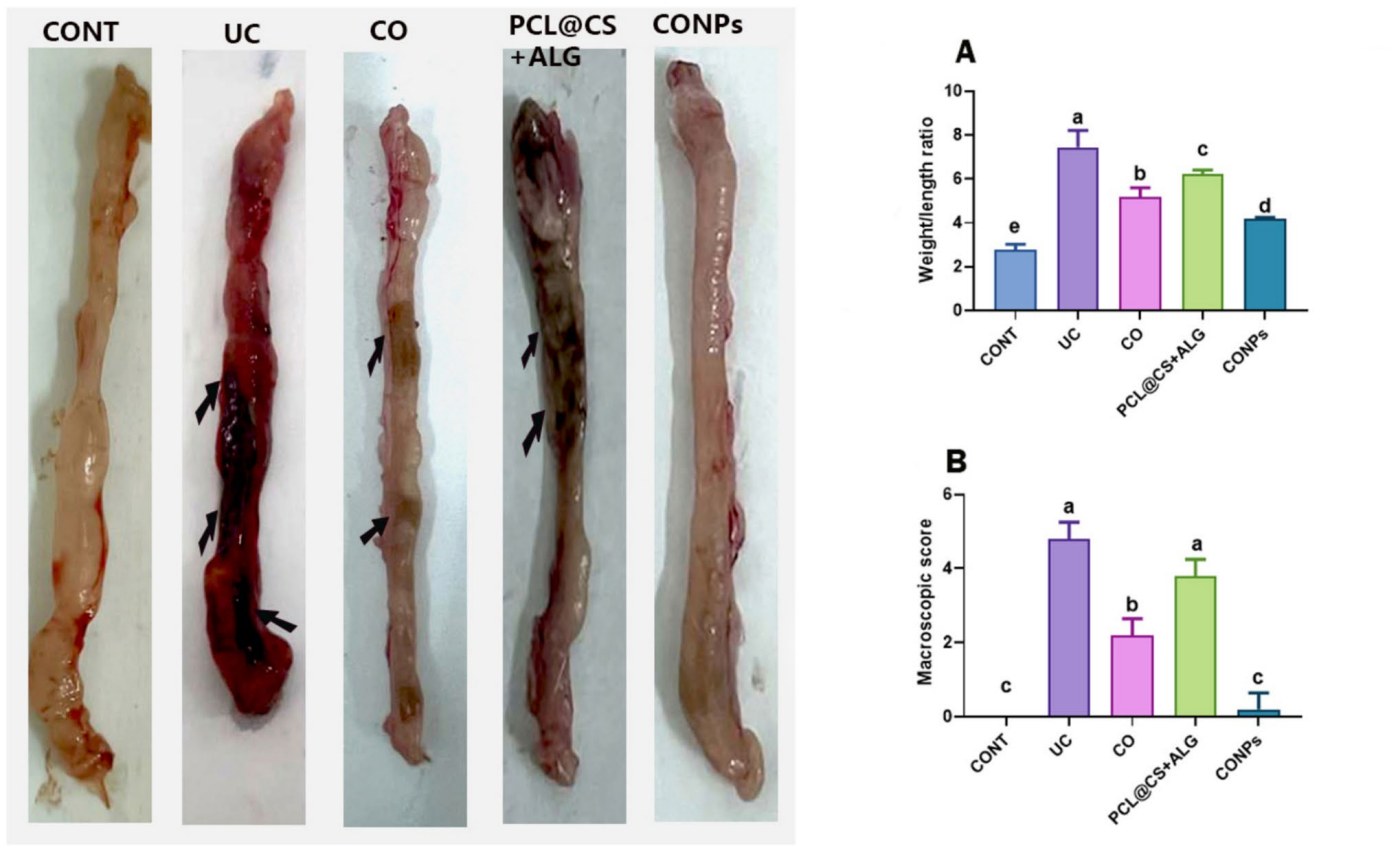
The macroscopic appearance of the ulcerative colitis score in all groups showed that the colon in the ulcerative group (UC) appeared edematous and hyperemic. The ulcerative area (black arrow) was extended more than 2 cm in the ulcerative (UC) and PCL@CS + ALC groups. The ulcerative area (black arrow) was decreased in the clove (CO) group. **A** representing the mean macroscopic ulcerative colitis scores in all treated groups. **B** representing the mean weight/length ratio of the colon in all treated groups. Data are expressed as Mean ± SEM. The different letters indicate a significant difference (*P* < 0.05) between experimental groups

The mean weight /length ratio (a marker of tissue edema) of the colon from UC and PCL@CS + ALG groups was significantly increased (*p* < 0.05) compared to the other groups (Fig. 9A). The administration of clove oil alone (CO group) or clove oil with nanoparticles (CONPs group) significantly ameliorated the increase in the weight-to-length ratio. Still, the beneficial effect was obtained when the clove oil was applied to the nanoparticles (CONPs group). The colon of untreated rats appeared hyperemic and ulcerative (Fig. 9B), and the mean score of colitis was a significant increase (*p* < 0.05) from 0 score in the control (CONT) group to 4.8 ± 0.2 in the untreated (UC) group and to 3.8 ± 0.2 in the PCL@CS + ALG group. In the clove (CO) group, the mean score (2.2 ± 0.2) was significantly decreased (*p* < 0.05) compared to the UC group. The normal macroscopic appearance of the colon was redetected in the CONPs group, and the score (0.2 ± 0.2) was nearly returned to the control one.

#### Microscopic ulcerative colitis scores

**Fig. 10:**
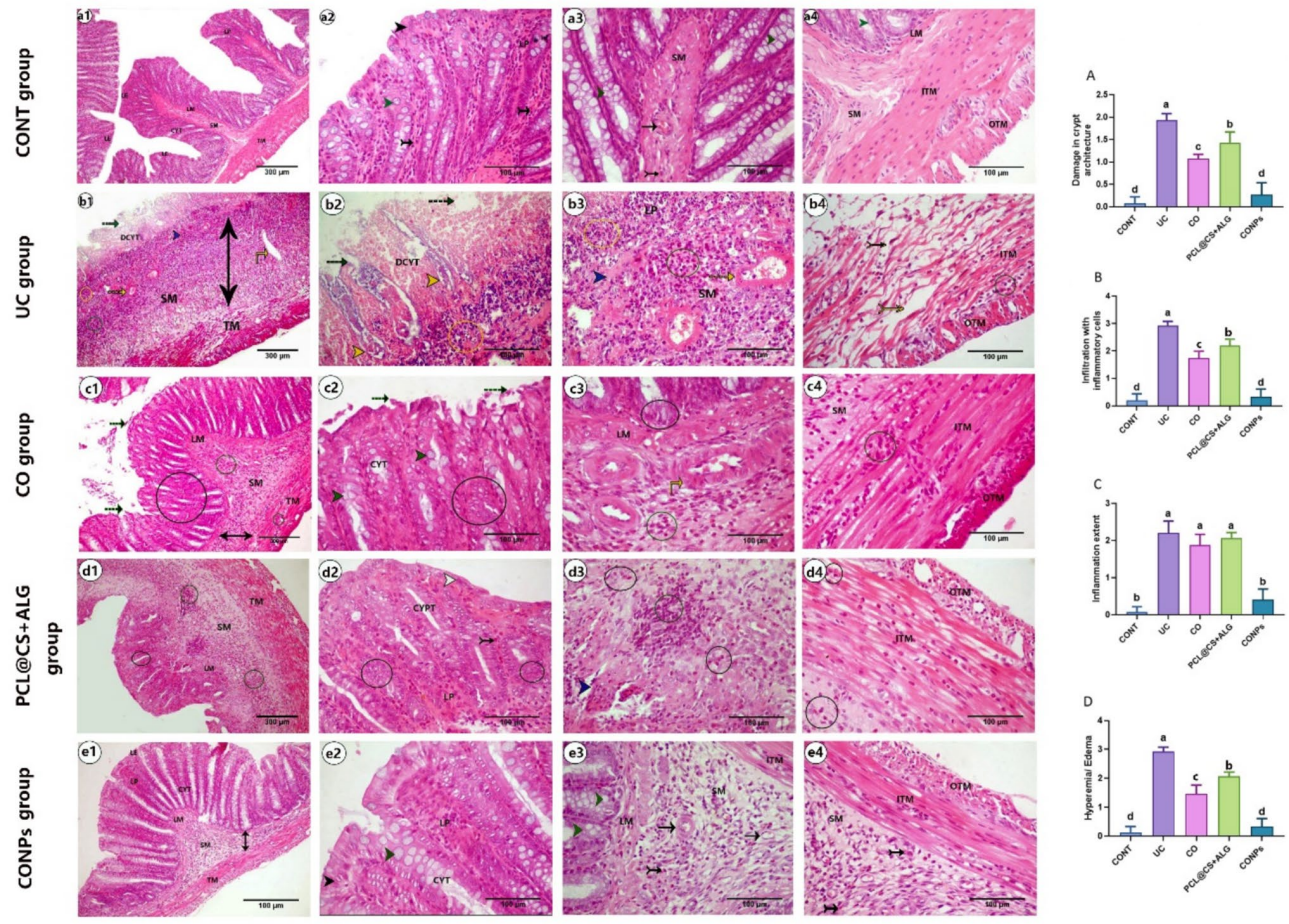
Photomicrograph of cross-sections of rat’s colon stained with H&E stain: showing lamina epithelialis (LE) lined with simple columnar epithelium (black arrow head), connective tissue of lamina propria (LP), normal colonic crypt (CYT) with normal goblet cells (green arrow head),( lamina musclaris (LM), connective tissues of submucosa (SM),blood vessels (black arrow),immune competent cells (tailed arrow),Tunica musclaris (TM) consisted of two layer inner tunica musclaris (ITM) and outer tunica musclaris (OTM),ulcerative lamina musclaris (discontinues green arrow),degenerated crypts(DCYT) filled with ulcer (yellow arrow head), crypt with abnormal shaped goblet cells (black circle), basal lymph plasmacytosis (yellow circle), Degenerated lamina musclaris (blue arrow head), inflammatory cells infiltration (green circles), edematous blood vessels (discontinues yellow arrow) and degenerated inner tunica musclaris (yellow tailed arrow), note double head black arrow represented thickness of submucosa. **A**–**D** representing the mean microscopic ulcerative colitis scores; **A** Crypt damage architecture score, **B** infiltration with inflammatory cells score, **C** inflammatory extent score, and hypermia/edema score. Data are expressed as Mean ± SEM. The different letters indicate a significant difference (*p* < 0.05) between experimental groups

The histological findings are represented in Fig. 10. The H&E-stained sections of the colon from the CONT group (Fig. 10a1–a4) exhibited normal histological architecture of the mucosa, which appeared continuous, compact, lined with simple columnar absorptive epithelium, interrupted with parallel-arranged tubular straight crypts lined with simple columnar epithelium and goblet cells. The intestinal crypts rested on a thin continuous layer of lamina muscularis and were embedded within a connective tissue layer of lamina propria. The narrow submucosal layer of connective tissues and blood vessels separates the mucosa from the inner circular and outer longitudinal layers of tunica muscularis. Regarding the UC group (Fig. 10b1–b4), it exhibits extensive and diffuse mucosal ulceration, with loss of the entire surface epithelium and crypts as indicated by the significantly increased crypt damage architecture score (Fig. 10A) compared to the control group (*p* < 0.05). A massive area of lymphocytes and plasma cell infiltrations was detected at the base of the ulcerative crypts (basal lymph plasmacytosis), and the lamina muscularis appeared attenuated. In addition, the submucosa was markedly expanded, primarily due to the extent of inflammation, infiltration with inflammatory cells, and edema of blood vessels. Scores (Fig. 10B–D) were significantly increased (*p* < 0.05) compared to the control group. The aggregation of mononuclear cell infiltration extended to the muscularis externa, causing degeneration and destruction of this layer. On the clove (CO) (Fig. 10C1–C4) and PCL@CS + ALC (Fig. 10d1–d4) groups, there is no significant difference in the inflammatory extent score between these two groups and the colitis group. In addition, the crypt damage architecture, inflammatory cell infiltration, and hyperemia/edema scores were significantly decreased compared to the colitis group; however, the improvement in these scores was faster in the clove oil groups than in the nano-control group. In the CONPs (Fig. 10e1–e4) group, all-colitis scores (Fig. 10A–D) were nearly returned to normal levels, similar to those of the control group, and the group exhibited relatively normal colon tissues with preservation of the surface epithelium and intestinal crypts.

#### Distribution of goblet cells

**Fig. 11:**
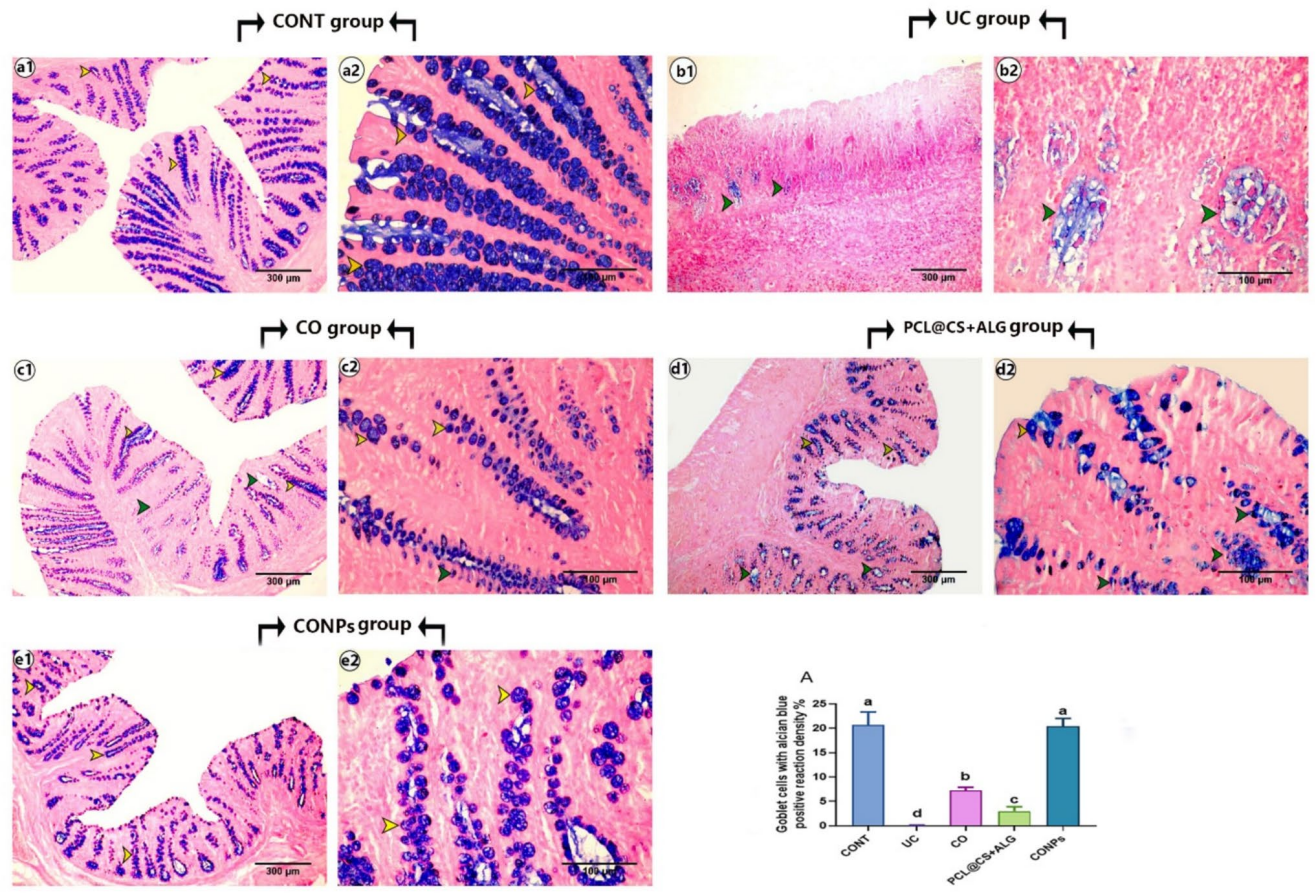
Photomicrograph of cross-sections of rat’s colon stained with Alcian blue: showing normal colonic crypt containing normal goblet cells with alcian blue-positive reaction (yellow arrowhead) and degenerated crypt with alcian blue negative reaction (green arrowhead). A represents the mean density % of goblet cells with an alcian blue-positive reaction. Data are expressed as Mean ± SEM. The different letters indicate a significant difference (*P* < 0.05) between experimental groups

In the CONT group (Fig. 11a1, a2), many goblet cells with positive reaction for alcian blue were detected on the colonic mucosa. In the ulcerative (Fig. 11b1, b2) group, the mean density of Goblet cells with positive alcian blue reaction was significantly decreased (Fig. 11A) compared to the control group (*p* < 0.05). There is an increase in this density in all treated groups (Fig. 11c1, c2, d1, d2, e1, and e2) compared to the colitis group. Still, the normal mean of alcian blue-positive goblet cells was detected only in the CONPs (Fig. 11e1, e2) group.

### CONPs regulated the VEGF and *α*-SMA immunohistochemical expressions of acetic acid-induced ulcerative colitis

As mentioned above, the colitis group exhibited extensive and diffuse mucosal ulceration, characterized by loss of the entire surface epithelium and crypts. The immunohistochemical expression of *α*-SMA and VEGF was hardly detectable in the mucosa from this group.

#### VEGF immunohistochemical expression

**Fig. 12:**
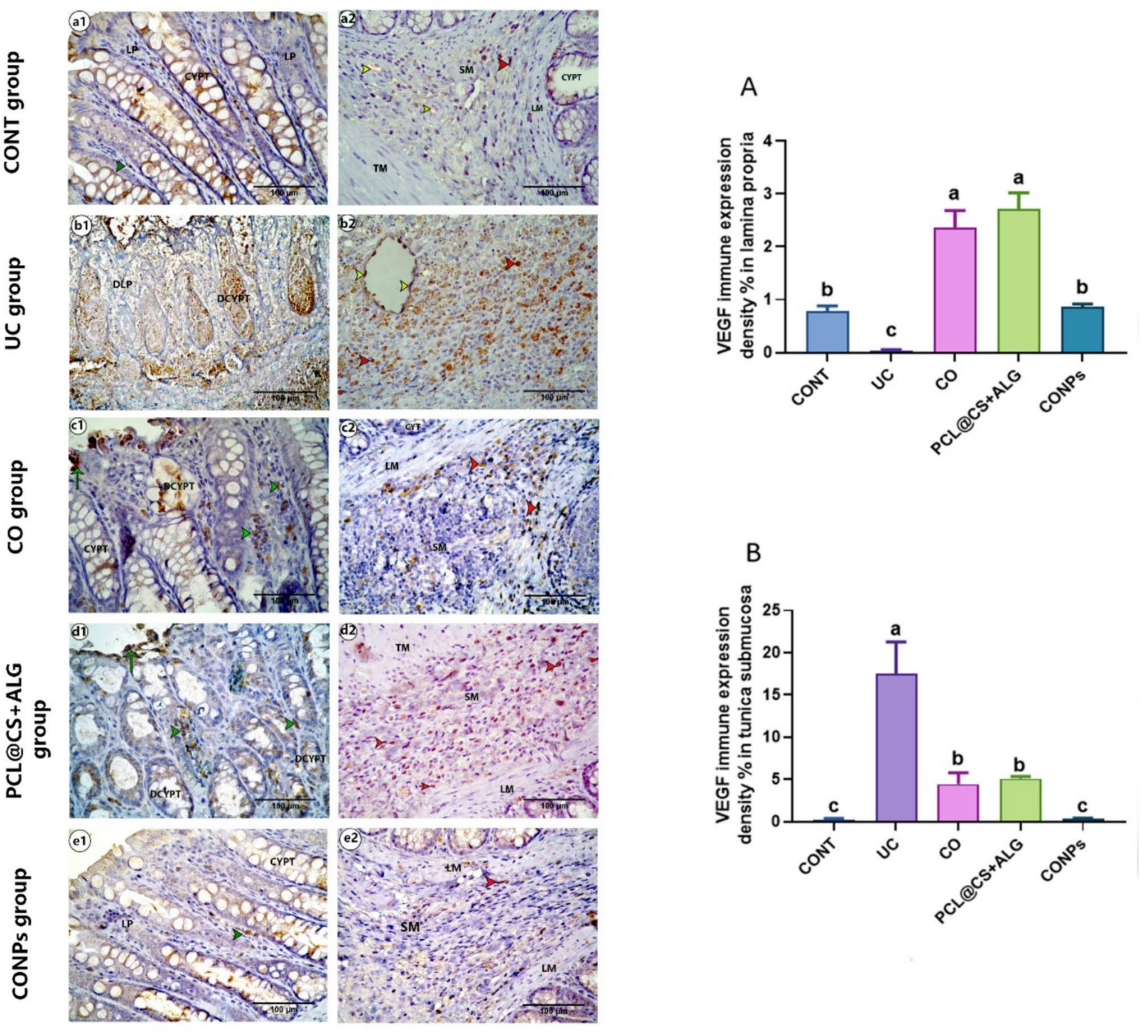
Photomicrograph of Enzyme immunohistochemical staining of paraffin sections from a colonic tissue for VEGF immunohistochemical expression showing cell with VEGF positive immune expression in lamina propria (LP) (green arrowhead) and submucosa (SM) (red arrowhead), endothelium of blood vessels with VEGF positive immune expression (yellow arrowhead), normal crypt (CYPT), degenerated crypt (DCYPT) and degenerated lamina propria (DLP). **A**, **B** representing the mean density % of cells with VEGF immune positive reaction in lamina propria and submucosa reprehensively. Data are expressed as Mean ± SEM. The different letters indicate a significant difference (*P* < 0.05) between experimental groups

The immune expression of the VEGF was only detected in the endothelium of the blood vessels in the control group. On the lamina propria, the mononuclear inflammatory cells with positive VEGF immune expression were detected in clove (Fig. 12c1, c2) and PCL@CS + ALC (Fig. 12d1, d2) groups, and a significant increase (*p* < 0.05) compared to the control group (Fig. 12A). On the submucosa (Fig. 12B), the highest immune expression of mononuclear inflammatory cells with VEGF was detected in the colitis group (Fig. 12b1, b2), followed by clove (Fig. 12c1, c2) and PCL@CS + ALG (Fig. 12d1, d2) groups. On the CONPs (Fig. 12e1, e2) group, the expression of VEGF was returned to the normal level.

#### α-SMA immunohistochemical expression

**Fig. 13:**
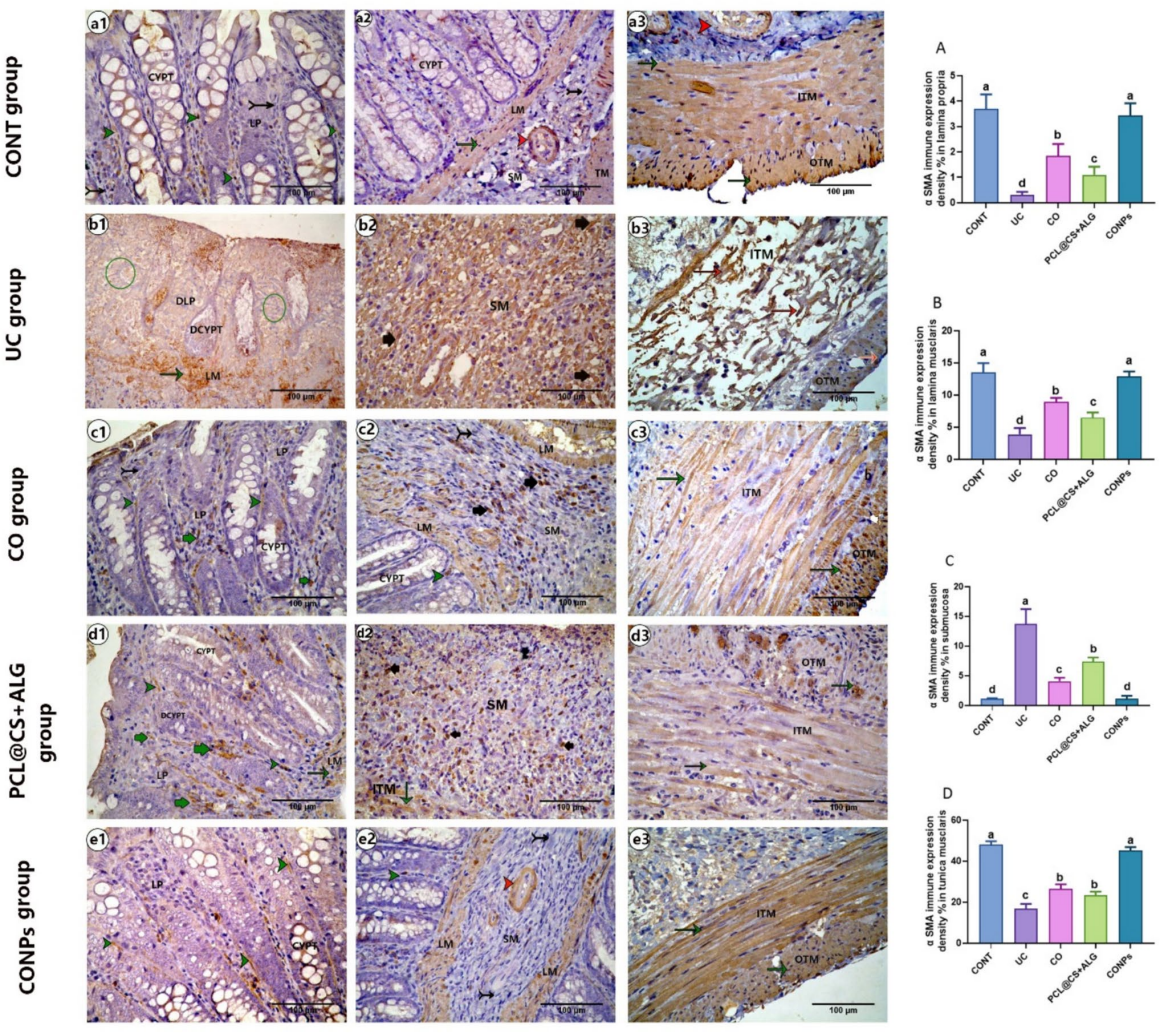
Photomicrograph of Enzyme immunohistochemical staining of paraffin sections from a colonic tissue for *α* SMA immunohistochemical expression showing peri-cryptal (subepithelial) myofibroblast with positive *α* SMA immunohistochemical expression (green arrowhead) in lamina propria (LP), CT fibroblast with negative *α* SMA immunohistochemical expression (black-tailed arrow), smooth muscle with positive *α* SMA immunohistochemical expression (green arrow) in lamina musclaris (LM) or tunica muscularis (TM) or in wall of blood vessels (orange arrowhead), degenerated lamina propria (DLP) devoid without any positive cells with *α* SMA immunohistochemical expression (green circle) and interstitial myofibroblast with positive *α* SMA immunohistochemical expression in lamina propria (thick short green arrow) or submucosa (thick short black arrow). Normal crypt (CYPT), degenerated crypt (DCYPT), inner tunica musclaris (ITM), and outer tunica musclaris (OTM). (A, B, C, and D) representing the mean density % of cells with *α* SMA immune positive reaction in lamina propria, lamina musclaris, submucosa, and tunica musclaris reprehensively. Data are expressed as Mean ± SEM. The different letters indicate a significant difference (*P* < 0.05) between experimental groups

On the mucosa of all groups, the immune expression of *α*-SMA of the CONT (Fig. 13a1, a2) group was detected only in the peri-cryptal (subepithelial) myofibroblast meanwhile in the clove oil (Fig. 13c1, c2) and PCL@CS + ALG (Fig. 13d1, d2) groups, the interstitial myofibroblasts with *α* SMA-positive immune expression was also detected in the lamina propria beside the peri-cryptal myofibroblast. Their expression was significantly (*p* < 0.05) increased compared to the control group. On the CONPs (Fig. 13e1, e2) group, the *α*-SMA immunohistochemical expression was nearly the same as that of the control group (Fig. 13A). On lamina muscularis (Fig. 13B), the highest expression of *α*-SMA was detected in the CONT and CONPs groups, followed by the CO and PCL@CS + ALG groups. The lowest expression of it was detected in the UC group. On the submucosa (Fig. 13C) of the control, the expression of *α*-SMA was detected only in the wall of the blood vessels; meanwhile, in the colitis (Fig. 13b1, b2), clove (Fig. 13c1, c2), and PCL@CS + ALG (Fig. 13d1, d2) group, the expression of it was detected in the interstitial myofibroblast and their immune density expression was significantly increased (*p* < 0.05) compared to the control group. The highest expression of it was detected in the UC group, followed by the nano-control, and then the clove group. In the CONPs group, the expression of *α*-SMA was nearly the same as in the control group. On the tunica muscularis (Fig. 13d), the highest expression of *α* SMA was detected in the CONT and CONPs groups, followed by the CO and PCL@CS + ALG groups, and then the UC group.

#### Multivariable data analysis

**Fig. 14:**
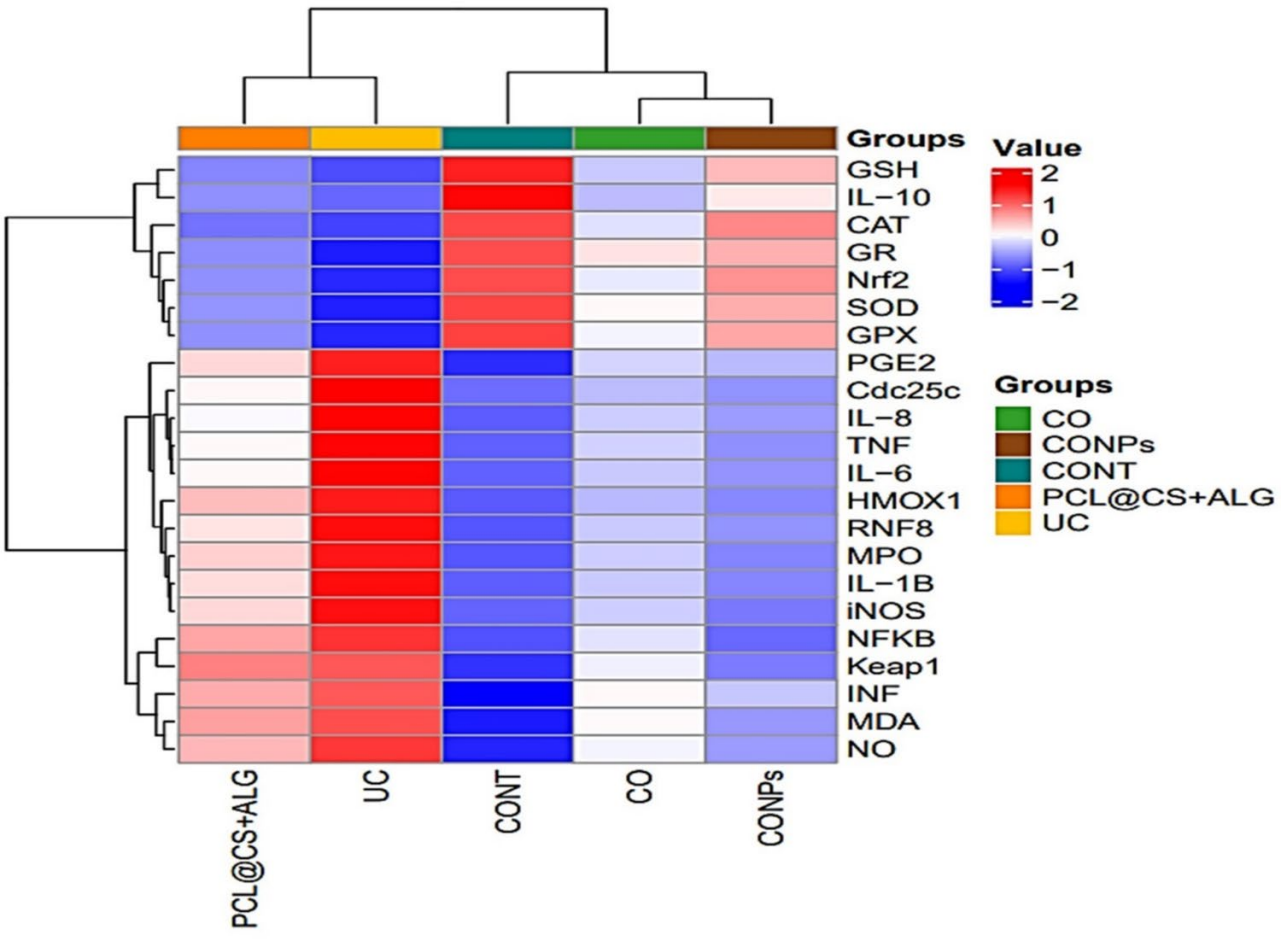
Hierarchical clustering heatmaps of biomarker profiles in experimental groups. Heatmap of oxidative stress and inflammatory markers across experimental groups (CONT: Control, Model of ulcerative colitis (UC), UC models treated with CO, PCL@CS + ALG, and CONPs

A comprehensive multivariable analysis of antioxidant and inflammatory markers in control (CONT), UC, UC treated with CO, PCL@CS + ALG, and CONPs treated with both was conducted further to assess the impact of the treatment under study. The UC group exhibited higher levels of oxidative stress markers (MDA and NO) and inflammatory markers (NF-κB and iNOS), along with lower levels of antioxidant enzyme activity (SOD, CAT, GR, and GPx), as indicated by biomarker hierarchical clustering heatmaps. On the other hand, the CONPs group exhibited biomarker levels that were more in line with the control group, indicating synergistic protection against UC. Additionally, specific parameters were partially standardized by the CO group, demonstrating the advantages of CO (Fig. 14).

## Discussion

The prevalence of UC continues to rise worldwide, accompanied by increasing medical and social expenses. The projected yearly direct and indirect expenditures associated with UC in the United States alone range from $8.1 billion to $14.9 billion (Cohen et al. 2010). In addition, the prevalence of UC has been steadily increasing (Ng et al. 2017), particularly in Asia, where it was previously low (Ng et al. 2019). The fact that chronic UC raises the risk of colon cancer (Dulai and Jairath 2018) and can be fatal if left untreated, which makes this alarming (Chu et al. 2017).

Chronic conditions like UC are thought to be influenced by oxidative stress (Shen et al. 2020). The present investigation found that AA significantly increased MDA and NO levels. This could be because active neutrophils and macrophages created nitric oxide, which in turn caused lipid peroxidation and the generation of excess MDA. El-Akabawy and El-Sherif (2019), Abdelaziz and Mahmoud (2023), and Rehman et al. (2024) reported similar results of high levels of MDA after injection of AA. Proinflammatory cytokines, such as TNF-*α*, activate iNOS, leading to increased nitric oxide production in UC. This increase promotes the generation of free radicals and attracts neutrophils and macrophages, thereby contributing to the pathophysiology of UC. Moreover, features, such as edema, erythema, inflammation, and diarrhea, are linked to nitric oxide levels in both serum and colon tissues (Kamalian et al. 2020). Colitis significantly decreases the levels of SOD and CAT, which have an essential protective response against oxidative stress (Abdelmegid et al. 2019; Rehman et al. 2024). Moreover, ROS production in the intestinal mucosa impairs blood flow, resulting in desquamation and loss of epithelial cells (Coskun 2014). Our results showed that colon tissue in the UC group had significantly higher levels of MDA, a commonly used indicator of lipid peroxidation, than in the control group. In contrast, the levels of GSH, GPx, and GR, as well as the enzymatic activity of the antioxidants SOD and CAT, were significantly lower in the UC group than in the control group. The low concentration of those antioxidant enzymes, which are primarily found in epithelial cells, may make the gut more vulnerable to oxidative damage (Cetinkaya et al. 2005). Our findings are also supported by earlier research (Badr et al. 2020; Rafeeq et al. 2021). Our histological results demonstrate that excessive generation of ROS and nitrogen metabolites does impact the intestinal mucosa's defense mechanism and eventually encourages the subsequent development of further inflammation and ulceration (Kruidenier and Verspaget 2002).

The shift in the expression pattern of the genes being studied may be caused by the acetic acid-induced colitis model, where inflammatory mediators, such as reactive oxygen species, vasoactive amines, and eicosanoids, play a significant role (Shahid et al. 2022). The underlying pathophysiological mechanisms involve disruption of blood clotting, elevated inflammatory mediators, increased vascular permeability, damage to colon structure, and mucosal barrier breakdown caused by chemical stimulation, which promotes fibrin hydrolysis. This creates a vicious cycle that generates more reactive metabolites, depletes cellular antioxidants, and further promotes inflammation and ulceration. An increase in lipid peroxides in colonic tissue can initiate this cycle (Thippeswamy et al. 2011). During inflammation, cells generate ROS and reactive nitrogen species (RNS), which are highly reactive molecules capable of damaging proteins, lipids, and nucleic acids within cells. In the gastrointestinal tract, oxidative stress additionally promotes the increased expression of genes associated with immune responses (Pravda 2005; Alzoghaibi 2013). In the current study, the macroscopic appearance of the colon in the model group, injected intrarectally with 1.5 ml of AA (5%), displayed hyperemia and ulceration, with a significantly increased mean score of colitis (4.8 ± 0.2) compared to the control group. The exact etiology of UC remains unknown; several processes are known to be involved in its pathogenesis (Graham and Xavier 2020). In this study, UC is primarily characterized by the initiation of oxidative stress, depletion of antioxidants, and increases in pro-inflammatory markers. Moreover, in this context, the UC group shows an increase in NO and PGE levels in colonic tissue. Sheibanie et al. (2007) noted that in DC, PGE2 inhibits the production of IL-12/IL-27 and promotes the expression of IL-23. In vivo, treatment with PGE analogs leads to the expansion of Th17 cells in the inflamed gut and elevated levels of IL-23 and IL-17.

NF-*κ*B and TNF-*α* are key pro-inflammatory mediators involved in the pathophysiology of UC. When a damaging stimulus occurs, TNF-*α* is released, subsequently activating the NF-*κ*B pathway. This activation leads to the inflammatory cascade by increasing the production of inflammatory mediators (Salama et al. 2021). TNF-*α* stimulates macrophages and neutrophils in the colon wall. This causes widespread intracellular acidification, resulting in acetic acid-induced colitis that damages mucosal barriers, injures the colonic epithelium, and triggers the release of inflammatory cytokines, which promote the recruitment of monocytes and macrophages (Jang et al. 2023). According to Shapiro et al. (2007), inflammatory cytokines (NF-*κ*B, IL-6, IL-1*β*, and iNOS) release several chemokines, which in turn encourage chemotaxis. Antioxidants protect by removing ROS from the environment, blocking their synthesis, or shielding transition metals needed for free radical production (Masella et al. 2005). Histological sections of the colon revealed inflammation that was parallel and confirmed by increased levels of pro-inflammatory markers in the serum of the UC group. It is well-known that TNF-α promotes the production of chemical mediators, proteases, and pro-inflammatory factors, which then induce chemotaxis and infiltration of inflammatory cells. Furthermore, it facilitates neutrophil infiltration by increasing the levels of adhesion molecules, leading to tissue damage (Muthas et al. 2017). Moreover, IL-6 has been linked to the severity of UC patients' disease and is essential for pro-inflammatory processes and immune function dysregulation (Bernardo et al. 2012), which can result in ulceration and bleeding by producing cytotoxic reactive oxygen species (Jainu et al. 2006; Munakata et al. 2003). As anticipated, we found that AA treatment significantly increased the levels of all pro-inflammatory cytokines and mediators, including TNF-*α*, IL-10, and IL-1*β*, in this investigation. Similarly, earlier research by Gu et al. (2017), Impellizzeri et al. (2018), and Zhu et al. (2019) showed that the colitis model significantly increased TNF-*α*, IL-1, and IL-17. Furthermore, following AA injection, Badr et al. (2020) noted a substantial increase in TNF-*α*, IL-1, IL-6, and IL-17.

The COX-2 enzyme is a key element in the complex network of biochemical mediators involved in UC's pathophysiology. It produces PGE2, which helps regulate apoptotic mediators and pro-inflammatory cytokines and may also play a role in the malignant consequences of UC. Our study found that COX-2 levels are increased in the inflamed colon (Kjærgaard et al. 2020). By encouraging neutrophil and macrophage infiltration into the colon wall, NF-*κ*B is one of the central pro-inflammatory mediators that drives inflammation in UC (Laurindo et al. 2023). Additionally, NF-*κ*B through IL-1*β* increases the expression of the COX-2 enzyme, which is previously known to play a role in inflammation (Chiba et al. 2012).

Neutrophils contain the enzyme myeloperoxidase, whereas monocytes and macrophages have considerably lower levels of this enzyme. Consequently, MPO activity is a quantitative and sensitive assay for acute intestinal inflammation and is directly correlated with the neutrophil concentration in the inflammatory region (Choudhary et al. 2001). In untreated rats, the colon exhibited elevated MPO levels, suggesting neutrophil infiltration and extracellular MPO release. This process contributes to mucin degradation, enhances mucosal permeability, and harms epithelial cells by catalyzing the oxidation of chloride with hydrogen peroxide, which generates hypochlorous acid, a reactive compound oxidant. These findings align with those reported by Cetinkaya et al. (2005), Kruidenier and Verspaget (2002), and Rafeeq et al. (2021). Histological colon sections showed thickened submucosa due to inflammation, cell infiltration, and edema, confirming the UC model. Mononuclear cells caused degeneration of the muscularis externa. Fibroblasts, leukocytes, and epithelial cells secrete VEGF, which promotes angiogenesis and increases vascular permeability (Bousvaros et al. 1999). In certain patients with colitis, VEGF was produced from epithelial mucosal cells (Taha et al. 2004).

Nrf2 is a crucial transcription factor that acts as an antioxidant. During oxidative stress, it translocates to the nucleus and activates, enhancing the production of various antioxidant enzymes downstream (Deng et al. 2022). It has been found that Nrf2 overexpression improves UC (Tan and Zheng 2018). The central inducible defense against oxidative stress is the Keap1-Nrf2 stress response system, which controls the production of cytoprotective genes (Baird et al. 2020). Under typical circumstances, the cullin-based E3 ubiquitin ligase, which impairs Nrf2 transcriptional activity through ubiquitination and proteasomal degradation, utilizes Keap1 as a substrate adapter (Kobayashi et al. 2004). This could account for the different expression patterns revealed by our analysis for the Keap1 and Nrf2 genes. The rate-limiting enzyme in the heme catabolic pathway is heme oxygenase (HO-1), which breaks down heme into equimolar quantities of free iron, biliverdin, and carbon monoxide (Consoli et al. 2021). It is recognized as a stress-responsive protein with anti-inflammatory, anti-apoptotic, and antioxidant properties, and it is hypothesized to carry out several protective functions against diverse stresses (Mohamed et al. 2022). Threonine/tyrosine phosphatase, which is involved in cell cycle regulation, is encoded by Cdc25c (Cho et al. 2015). It controls the transition from the G_2_ to the M phase of the cell cycle, blockage results in cell cycle arrest and increased apoptosis. DNA repair involving non-homologous end joining, homologous recombination, and nucleotide excision is carried out by RNF8 (Lu et al. 2012). According to Aichinger et al. (Aichinger et al. 2022), the Alternaria toxin causes the rat colon to become positive for the DNA damage markers Cdc25c and RNF8. In the current study, rats exposed to acetic acid had their expression patterns for NFKB, IL-6, IL-8, and iNOS, as well as for antioxidant (Nrf2, Keap1, and HO-1) and DNA damage (Cdc25c and RNF8) genes, evaluated. Compared to the control group, our findings showed that the genes NF-*κ*B, IL-6, IL-1*β*, iNOS, Keap1, HO-1, and Cdc25c were up-regulated. Nrf2 mRNA levels, on the other hand, decreased. According to Shahid et al. (2022), acetic acid increases the expression patterns of apoptotic (Bax and caspase-3) and inflammatory (TNF-*α*, IL-6, MPO, PGE2, COX-2, and NF-*κ*B) indicators. But there was a decrease in the anti-apoptotic protein (Bcl-2). Furthermore, compared to the control group, rats treated with acetic acid showed a significant increase in colonic TLR-4 gene expression (Alanazi et al. 2023). Fawzy et al. (2022) also mentioned that in colitis caused by acetic acid, the expression profile of the IL-1*β* gene is up-regulated.

Poor treatment outcomes and the frequency of adverse effects have created a need for novel, practical therapeutic approaches (Yuan et al. 2006; Clark et al. 2007). As current immunosuppressants, sulphonamides and hormone agents used for the treatment of UC only control the UC symptoms, and unfortunately, induce side effects and hormone resistance (Gao et al. 2019). Recently, plant-based drugs have been gaining more attention due to their potential effect in UC (Liu et al. 2021; Patel et al. 2022). Besides having limited side effects, they are increasingly being utilized for the treatment of IBD due to the beneficial effects of their antioxidants on experimental colitis (Nosál'ová et al. 2000). This is the first study to compare the potential protective and therapeutic effects of clove oil versus clove oil coated with CS and alginate nanoparticles. In the current study, pretreatment with CO or CONPs exhibits powerful antioxidant and anti-inflammatory effects, thereby mitigating the occurrence of UC.

To the best of our knowledge, a limited number of studies have been conducted on the effects of essential oils on in vitro cell migration. In addition, CO's high volatility and susceptibility to deterioration from exposure to oxygen, heat, and light limit its use (El Asbahani et al. 2015). Various preparation techniques have been employed to create CO polymer carriers, including nanoparticles, to increase the application of CO (Alam et al. 2017; Cui et al. 2018). There are benefits to using nanoemulsions, including improved delivery efficacy, physical stability, and resistance to volatility and degradation (Singh et al. 2009). The CS-induced mucoadhesion effect was confined to the surface of each droplet. Due to the droplets' nanometric size, which resulted in an enhanced surface-to-volume ratio, the encapsulated oil was able to make greater contact with the mucosal surface. By improving the process of passive transport, pharmaceuticals enclosed in emulsions enable the absorption of medicinal ingredients with increased particle uptake. Additionally, nanoemulsions are anticipated to pass through biological barriers without encountering any obstacles (Alzorqi et al. 2016). For NPs loaded with CO to be transported to the colon's inflammation site and enhance epithelial permeability and retention effect (Xiao et al. 2017), biomaterials composed of ALG and CS can be broken down in the colon without being harmed by the gastric environment (Zhang et al. 2018).

Originally native to Asia, *S. aromaticum* is recognized for its high content of phenolic compounds. Cloves have significant potential in food, medicine, cosmetics, and agriculture. Due to its medicinal properties, the essential oil derived from clove is extensively utilized. (Ramadan et al. 2013). It contains eugenol, phenols, flavonoids, oleanolic acid, and tannins, which have been demonstrated to reduce oxidative stress-induced damage to the brain, liver, heart, kidneys, and testicles (Abtahi-Eivari et al. 2021; Barghi et al. 2021). In recent years, essential oils derived from various plant sources have been used to treat a wide range of illnesses. It has been stated that using CO, which is produced from the dried buds of *S. aromaticum*, can reduce pain and aid in the healing process (Chaieb et al. 2007). Eugenol, a primary phenolic component of CO, has been linked to several critical pharmacological advantages, including anti-inflammatory, antibacterial, and antifungal characteristics (Kalemba and Kunicka 2003). Given that CO is highly volatile, efforts were made to encapsulate phenolic CO in delivery vehicles that are nanoscale, biocompatible, and biodegradable (Anwer et al. 2014).

The uniform spherical nano-PCL@CO formulations produced by the emulsion-evaporation technique have diameters that progressively increase with increasing CO supplementation. Despite this, the CO coated with ALG/CS embedded through PCL had consistent particle distribution. A new peak emerged in the nano-formulations upon the addition of CO. According to Liverani and Boccaccin (2016), peaks corresponding to carbonyl (C = O) stretching and both symmetric and asymmetric stretching of C–O–C bonds were found at 1725.805, 1244.137, and 1166.250 cm^−1^, respectively. Additionally, in earlier research, distinctive FT-IR peaks representing the C–C aromatic ring vibrations of eugenol were observed at 1637 cm^−1^, 1606 cm^−1^, and 1513 cm^−1^ (Yadav and Saini 1994; Smith et al. 1988; Mohammed et al. 2016). As explained by Smith (1988) and Keawpeng et al. (Keawpeng et al. 2022), the stretching vibration of C = C aromatic bonds is responsible for the CO bands that were detected at 1514 cm^−1^, which corresponds to the primary component of CO—eugenol. The GC–MS analysis was used to confirm the presence of the appropriate components in the clove oil. These peaks were retained, and the CO nano-formulations exhibited a discernible peak shift, indicating that the CO and nanoparticles were indeed present in the matrix. The interactions between the constituent parts of the bionanocomposite may explain the apparent shift of these peaks. Natural nanoparticle clustering was observed in SEM images; this is most likely the result of operations where the surfactant was not present, leading to inadequate steric stabilization. Drug carriers with a diameter of less than 400 nm are confirmed to be the ideal size for administering UC drugs. Smaller NPs (less than 400 nm) can therefore be utilized as UC delivery vehicles, with a high potential to enhance the effectiveness of UC treatment. Moreover, particle size affects the process of particle absorption by cells. Because PCL polymer has carboxylic end groups (Öcal et al. 2014) and anionic biopolymer ALG contains carboxylic and hydroxyl end groups, it is expected that all created NP formulations will have a negative surface charge. Conversely, when coating with the CS, the zeta potential values changed to positive ones. The amino groups in CS, which were present on the surface of the nanoparticles, cause the negative zeta potential to change into a positive one. Therefore, having a charge that may readily engage through an ionic adsorption process with the nexus charged cell membrane may result in increased cellular absorption. Polysaccharide-based NPs, including CSNPs, are known to shrink or lose form when exposed to electron bombardment during SEM investigation, in contrast to polymeric nanoparticles like PCL and PLGA.

After being suspended in water (w/v 2/1) for a short while, the nanoparticles were ultrasonically agitated. The infiltrative ability of the generated nano-PCL@CO formulations has been significantly enhanced using water as a potential nanoparticle carrier vehicle, which is crucial for effective particle penetration. The percentage of EE is substantially impacted by the degree of CO solubility in the material. Furthermore, the emulsion-evaporation process typically results in high encapsulation values for hydrophobic substances. Considering this, a maximum %EE of 96.85% and a %LC of 20.39% were attained. According to the absorption spectra of essential CO, the electronic transition from the type *n–π for the alcohol group that binds with the aromatic ring, * n–π of the group carbonyl binds with the aromatic ring of quinone, and * π–π of the group ketone that binds with the aromatic ring and gives these compounds their yellow color occurred at 210, 227, and 282 nm, respectively (Nahar and Sarker 2019). This demonstrated the presence of clove's primary bioactive ingredient, eugenol. Therefore, using alcohol as a blank, the absorbance of CO was measured at 282 nm.

The release of CO close to the surface of nano-PCL@CO formulations caused the in vitro cumulative CO-release to follow a biphasic pattern, with an initial fast CO-burst lasting 4 h (Sansdrap and Moës, 1997). According to earlier descriptions, CO, especially surfactant-like eugenol, has an attraction for surfactant molecules, which may cause some of it to migrate to the surface of the nanoparticle (Tchakalova et al. 2008). PCL's carboxyl groups may have reacted with CO's OH groups, including eugenol, during encapsulation (DeRuiter, 2005).

Over time, the medicine can be released from nanoparticles because this phenomenon is reversible. As a result, after 120 h, a continuous progressive release of 29%, 33.5%, 41.5%, and 48% of CO was seen, caused by a combination of hydrolysis, degradation, and polymer diffusion (Sahana et al. 2008). The retention of the remaining released CO in the PCL matrix may be the cause of the emission of such small amounts. Although the CO-release profile in this study was only measured for 120 h, the steadier, longer release pattern suggests that CO will likely be emitted for a more extended period. The presence of the adsorbed drug on the surface of nanoparticles containing excess amounts of necessary CO, which were quickly dissolved into the medium from the matrix PCL, may have caused the drug's first burst release. The most crystalline and hydrophobic polymers degrade at the slowest rate (Lemoine et al. 1996; Lamprecht et al. 2000). Despite being hydrophobic as well, vital CO was released far less from PCL nanoparticles. According to some researchers, the gradual breakdown of polymers in the release media may cause release from these polymers to last for several months (Malhotra and Majumdar 2001). The burst effect's strength and the total rate of CO release were both decreased by the CS and ALG coating. The existence and function of the outer ALG layer as an additional barrier for CO release may help explain this. To further limit drug release, CS and ALG can interact with biological fluids, salts, and various delivery media to which formulations are exposed. Because CS offers a closed layer of PCL@CONPs, which serves as a physical barrier to the diffusion of CO into the bulk solution, the slow release of CO may be explained. In the past, PCL-based NPs have been used to extend the release pattern of numerous drugs (Coffin and McGinity 1992). The drug connection near the particle surface, and upon contact with the dissolving solvent, may be the cause of the initial burst release of the drug from the nano-formulations. On the other hand, the drug's regulated release from the nano-formulations may be caused by the polymeric shell's obstructive effects and increased diffusion distance (Kalimouttou et al. 2009; Patel et al. 2014).

An *in silico* study revealed the molecular interaction abilities of eugenol, the most abundant bioactive compound of *S. aromaticum*, with NF-*κ*B, IL-6, iNOS, Keap1, and HO-1 proteins. This study is a molecular screening conducted before an in vivo study. Administration of CO or CONPs attenuates oxidative stress induced by acetic acid. In the rat group with CO, a significant decline in colonic levels of MDA and NO was observed when compared to the untreated groups. Additionally, pretreatments restore the antioxidant equilibrium by inducing a considerable increase in the levels of SOD, catalase, GSH, GPx, and GR compared to the UC group and the carrier group. Notably, the glutathione group is involved in the synthesis and repair of DNA, blocking free radical damage (Chavan et al. 2005). Our findings are in correspondence with the studies of Hassanen (2010) and Soltani et al. (2023), which revealed a decline in MDA levels and an increase in GPx activity and GSH after administration of clove oil in diabetic and PCOS rats, respectively. This may be related to the high content of eugenol derivatives in clove oil. As an antioxidant, eugenol effectively inhibits protein denaturation, which may maintain the integrity of the cell membrane and prevent intracellular enzyme leakage (Ulanowska and Olas 2021), thereby illuminating the integrity of colon tissue in treated groups, especially those pretreated with CONPs.

Clove oil or in nano-form diminishes infiltration of large numbers of phagocytic leukocytes into the mucosal interstitial, confirmed microscopically in histological sections of treated groups (CO and CONPs) as compared to UC group in the current study, reflecting powerful anti- inflammatory effect of CO. This may also have attributed to eugenol derivatives compound, it has anti-inflammatory effect, and prevent prostaglandin synthesis. Our findings revealed a significant reduction in all measured inflammatory markers and mediators of the CO and CONPs groups when compared to UC, including MPO, TNF-*α*, IL-6, IL-1*β*, and IL-10. This confirms less infiltration of colon tissue by inflammatory cells. Moreover, PGE levels declined sharply in groups treated with clove CO or CONPs compared to the untreated UC group. Prostaglandins have been demonstrated to promote the production of inflammatory exudates during tenderness. Taher et al. (2015) found that clove oil has anti-inflammatory properties, reducing edema production three hours after carrageenan injection. This suggests that the oil's effects are likely mediated by interfering with the production of prostaglandins.

An *in silico* approach revealed that eugenol exhibited high binding affinities *to NF-κB, IL-6*, *IL-1β*, *iNOS*, *Keap1*, *HO-1*, and *Cdc25c*, which may consequently lead to a decrease in their expression. The gene expression approach was employed to investigate the ameliorative effects of clove oil, PCL@CS + ALG, or clove oil nanoparticles in acetic acid-induced colitis. Rats exposed to clove oil, PCL@CS + ALG, or clove oil nanoparticles elicited a significant downregulation of NF-*κ*B, IL-6, IL-1*β*, iNOS, Keap1, HO-1, Cdc25c, and RNF8. However, Nrf2 genes were significantly up-regulated, as reported by Jabbari et al. (2020), who stated that eugenol encapsulated in CS nanoparticles reduces TGF-*β* gene expression in an aggressive form of rheumatoid arthritis. According to earlier research, clove oil acts as an anti-inflammatory on COX-2 and 5-LOX, altering the NF-*κ*B pathway and reducing the synthesis of interleukin-6 (Karaji et al. 2023). By affecting the NF-*κ*B pathway and interleukin-6 synthesis, which are crucial elements of inflammation, clove oil appears to reduce NLRP1 mRNA and protein expression (Wie et al. 1997). Research has demonstrated that the main component of clove oil, eugenol, essentially possesses immunomodulatory, DNA-protective, anti-genotoxic, antioxidant, and anti-inflammatory properties (Yogalakshmi et al. 2010; Sharma et al. 2011). Because it inhibits prostaglandin synthesis and suppresses neutrophil/macrophage chemotaxis, eugenol also has anti-inflammatory properties (Estevão-Silva et al. 2014). Furthermore, rheumatoid arthritis may be treated with CS nanoparticles, which Eugenol has identified as a potent antioxidant (Jabbari et al. 2020).

In general, the nano-formulation of clove oil was more effective in the prevention and treatment of UC than the natural form of the CO, where most of the parameters returned to normal levels. The co-treatment with CONPs restored colon catalase and GR activities to their normal values, like those of the control rats. Interestingly, the lower level of oxidative biomarkers and the higher level of antioxidant molecules were detected in rats treated with NCOPs. Additionally, only in the CONPs group does the number of goblet cells increase to the normal value. In comparison to the other groups, the higher goblet cell count was also linked to improvements in all colitis scores and intestinal mucosal regeneration. This could be explained by the fact that the encapsulated version of clove oil has more antioxidant activity than clove oil itself (de Oliveira and Tavares 2022). Because nanoparticles have larger surface areas than comparable masses of larger-scale materials, it may be possible to enhance the bioavailability and bio-effectiveness of essential oils. Additionally, this particle size makes it possible to lower the oil dosages required to provide antioxidant and antibacterial effects (Hossain et al. 2019; Tiwari et al. 2020). Additionally, the primary antioxidant in clove oil, eugenol, had its cytotoxic effect lessened by the addition of PLA nanoparticles (de Oliveira and Tavares 2022). This explains the superior effect of CONPs over natural oil in this study.

The model of UC in this study was confirmed through histopathological sections of the colon. In the current study, the ulcerative group exhibited extensive and diffuse mucosal ulceration, characterized by loss of the entire surface epithelium and crypts, as well as a significant increase in both the inflammation score and the inflammatory infiltration score compared to the control group. In addition, the submucosa was markedly expanded, primarily due to the importance of inflammation, infiltration with inflammatory cells, and edema of blood vessels. Scores were significantly increased compared to the control group. Previous investigators have also detected such results (Abd El-Galil et al. 2015; Lean et al. 2015; Amal et al. 2018).

A significant decrease in the number of goblet cells was also observed in the UC model of the current study. Goblet cells produce mucins to maintain the mucus barrier. It separates intestinal microbial flora from the epithelium and aids in immunological activities like antigen presentation and tolerance (Merga et al. 2014). Recent research has highlighted the role of the mucous barrier in IBD pathogenesis, which is accompanied by a deficiency in the differentiation of intestinal stem cells to goblet cells (Gersemann et al. 2009, 2011) and a deficiency in the intestinal mucous barrier. Only in the CONPs group was the number of goblet cells increased to the normal value. The increased number of goblet cells was concurrently associated with improvement of all colitis scores and the regeneration of the intestinal mucosa compared to the other groups.

VEGF is a cytokine produced by epithelial cells, leucocytes, and fibroblasts that promotes vascular permeability and enhances angiogenesis (Bousvaros et al. 1999). In certain patients with colitis, VEGF was produced from the epithelial mucosal cells (Taha et al. 2004). In the current study, the expression of VEGF in the mucosa of the colon from the colitis group was barely detectable, as the colitis group exhibited extensive and diffused mucosal ulceration, resulting in the loss of the entire thickness of the surface epithelium and crypts. On the other hand, its expression was significantly increased in the submucosa of the colon from the UC group, suggesting a role for mononuclear inflammatory cells in the submucosa in the secretion of VEGF, a finding similar to my own, as reported by Koch et al. (1986) investigated that activated macrophages are sufficient to secrete VEGF. About the role of VEGF in the pathogenesis of UC, VEGF increases the permeability of blood vessels and enhances the infiltration of inflammatory cells in the UC group (Tolstanova et al. 2010). The expression of VEGF was nearly returned to the normal level in the CONPs group, as was the mononuclear cell infiltration score.

Myofibroblasts are specific cells that share properties of smooth muscle cells and fibroblasts. Their ability to produce chemokines, cytokines, matrix components, and prostaglandins is believed to play essential roles in inflammation, growth, repair, and neoplasia (Adegboyega et al. 2002). Colonic myofibroblasts can be either subepithelial or interstitial. Subepithelial myofibroblasts are known as colonic stellate cells. These cells are located at the crypt base, between the mucosal epithelium and capillaries, and play a role in UC regeneration. Interstitial myofibroblasts, found throughout the lamina propria and submucosa, contribute to fibrosis (Okayasu et al. 2009, 2011). In the current study, immune expression was detected only in the myoepithelial fibroblasts of the control group and CONPs groups. The interstitial myofibroblast with positive *α*-SMA immune expression was detected in the submucosa of the UC group and lamina propria and submucosa of CO and PCL@CS + ALG groups (hardly detected in the mucosa of the colitis group as the colitis group exhibited extensive and diffused mucosal ulceration with loss of the entire thickness of surface epithelium and crypts. My findings are compatible with the results of Adegboyega et al. (2002), who demonstrated that subepithelial myofibroblasts with positive *α*-SMA immune expression were found in the normal colonic mucosa; meanwhile, the interstitial myofibroblast with positive *α*-SMA immune expression was detected in the colon of a patient with UC and significantly increased compared to the control colon (Andoh et al. 2002). In UC patients, there is an increase in interstitial myofibroblasts and a decrease in subepithelial myofibroblasts, which promote regeneration (Okayasu et al. 2009). Fibroblasts were activated into myofibroblasts under the effect of inflammatory cytokines (Stallmach et al. 1992). In the current study, the immune expression of *α*-SMA was assessed in parallel with the score of inflammatory cell infiltration in all groups.

In addition to that, *α*-SMA acts as a powerful protein in the identification of normal and pathological conditions of smooth muscle (Okayasu et al. 2009). *α*-SMA immune expression was also detected in the smooth muscle cells of the lamina muscularis and tunica muscularis of the colon. In the current study, the lamina muscularis appeared attenuated, and the tunica muscularis appeared degenerated in the UC group. Consequently, the *α*-SMA immune expression was significantly decreased in these layers compared to the control group. The normal histoarchitecture of the lamina and tunica muscularis was nearly detected in the CONPs group; therefore, the *α*-SMA immune expression was almost returned to the normal level in this group.

A limitation of the present study is that only a single dose level of clove oil and its nano-formulation (250 mg/kg) was tested, without the inclusion of a standard positive control drug. Although this dose was carefully selected based on OECD guidelines and previous toxicity data to ensure safety and biological efficacy, the absence of a dose–response design and therapeutic reference limits the full extrapolation of the findings. Future investigations will therefore be extended to evaluate multiple dose levels and to include a positive control (e.g., sulfasalazine or mesalamine) in order to comply with standard preclinical requirements and provide more comprehensive evidence of therapeutic efficacy.

## Conclusion

In conclusion, CO-loaded PCLNPs coated with CS/ALG (CONPs) significantly reduced oxidative damage and inflammatory mediators in the colon damaged by AA. Presumably, this protection may be due to: (1) CO and CONPs induced regulation of Keap1/Nrf2/HO-1 signaling pathway, which lessens damage to the colon mucosa; (2) CO and CONPs mediated inhibition of NF-*κ*B translocation to the nucleus, which stops the NF-*κ*B-dependent pathway; and (3) CO and CONPs generated suppression of free radicals and enhancement of endogenous antioxidant defense systems. These findings have crucially clarified the therapeutic potential of coating CO with chitosan/alginate nanoparticles to cure UC mediated by AA.

## Abbreviations

UC: Ulcerative colitis
CO: Clove essential oil
CONPs: Clove oil nanoparticles
ALG: Alginate
CS: Chitosan
PCL: Polycaprolactone
AA: Acetic acid
DMSO: Dimethyl sulfoxide
MDA: Malondialdehyde
NO: Nitric oxide
SOD: Superoxide dismutase
CAT: Catalase
GSH: Glutathione reduced
GPx: Glutathione peroxidase
GR: Glutathione reductase
MPO: Myeloperoxidase
PGE2: Prostaglandin
E2: TNF-*α*, Tumor necrosis factor-alpha
IFN-*γ*: Interferon gamma
IL-1*β*: Interleukin-1*β*
IL-10: Interleukin-10
NF-*κ*B: Nuclear factor kappa B
IL-6: Interleukin-6
IL-8: Interleukin-8
iNOS: Inducible nitric oxide synthase
Nrf2: Nuclear factor E2-related factor 2
Keap1: Kelch-like ECH-associated protein 1
HO-1: Heme_oxygenase 1
Cdc25c: Cell division cycle 25C
RNF8: Ring finger protein 8
LBL: Layer by layer
MWCO: Molecular weight cut-off
IBD: Inflammatory bowel disease
PLGA: Poly-lactic-co-glycolic acid

## Declarations

### Availability of data and material

The datasets used and/or analyzed during the current study are available from the corresponding author on reasonable request.
